# The immunoregulatory role of monocytes and thrombomodulin in myelodysplastic neoplasms

**DOI:** 10.3389/fonc.2024.1414102

**Published:** 2024-07-26

**Authors:** Luca L. G. Janssen, Nathalie van Leeuwen-Kerkhoff, Theresia M. Westers, Tanja D. de Gruijl, Arjan A. van de Loosdrecht

**Affiliations:** ^1^ Department of Hematology, Amsterdam University Medical Center (UMC), Vrije Universiteit, Amsterdam, Netherlands; ^2^ Cancer Center Amsterdam, Cancer Biology and Immunology, Amsterdam, Netherlands; ^3^ Department of Medical Oncology, Amsterdam University Medical Center (UMC), Vrije Universiteit, Amsterdam, Netherlands; ^4^ Amsterdam Institute for Immunity and Infectious Diseases, Amsterdam, Netherlands

**Keywords:** monocytes, thrombomodulin (TM), myelodysplastic neoplasms (MDS), immune regulation, inflammation

## Abstract

Myelodysplastic neoplasms (MDS) are clonal disorders of the myeloid lineage leading to peripheral blood cytopenias. Dysregulation of innate immunity is hypothesized to be a potent driver of MDS. A recent study revealed increased thrombomodulin (TM) expression on classical monocytes in MDS, which was associated with prolonged survival. TM is a receptor with immunoregulatory capacities, however, its exact role in MDS development remains to be elucidated. In this review we focus on normal monocyte biology and report on the involvement of monocytes in myeloid disease entities with a special focus on MDS. Furthermore, we delve into the current knowledge on TM and its function in monocytes in health and disease and explore the role of TM-expressing monocytes as driver, supporter or epiphenomenon in the MDS bone marrow environment.

## 1 Introduction

Myelodysplastic neoplasms (MDS) are a group of clonal myeloid stem and progenitor cell disorders, characterized by bone marrow (BM) failure resulting in cytopenias and a propensity to progress to acute myeloid leukemia (AML) (1, 2). Patients present mostly with fatigue, infections and/or bleeding disorders dependent on type and number of affected cell lineages. Also, autoimmune features may be observed, since there is an increased incidence of systemic inflammatory and autoimmune disorders (3). The higher prevalence of autoimmune diseases within MDS patients is correlated with a higher median overall survival (4, 5), suggesting superior immune surveillance. Furthermore, patients with a history of autoimmune disease have an increased risk of developing MDS (3). Therefore, immune dysregulation is hypothesized to be a potent driver in the pathogenesis of MDS (6).

Monocytes fulfill a crucial role in the immune system and their presence in MDS has been correlated to leukemic transformation (7). Interestingly, our group observed an increased percentage of thrombomodulin (TM)-expressing classical monocytes in BM as well as in peripheral blood (PB) (8). No differences were found regarding TM expression on intermediate and non-classical monocytes in MDS. TM-expressing classical monocytes were predominantly observed in low-risk MDS and correlated to a better overall and leukemia-free survival of MDS patients. These TM-expressing monocytes seemed to induce T cells with a more tolerogenic phenotype. Since TM has been linked to anti-inflammatory properties previously, we hypothesized that these TM-expressing monocytes could play an essential role in MDS pathogenesis and leukemic progression by regulating disproportional inflammatory responses (8). However, the exact mechanism remains to be elucidated.

To date, reviews on TM expression comprise mostly endothelial-TM (9, 10). Contradictions on function between TM-expressing monocytes and TM-expressing endothelial cells have been described (9), which underscores the importance of discriminating the cell type in which TM is expressed. In this review, we first describe current knowledge on monocyte biology in healthy aging individuals. Second, we elaborate on the role of monocytes in MDS. Third, we focus on studies that have described TM expression by monocytes and functional consequences thereof. Finally, we formulate hypotheses on the role of TM-expressing monocytes in MDS pathogenesis and progression as well as options for therapeutic intervention.

## 2 Monocyte biology in healthy, aging individuals

### 2.1 Monocyte ontogeny, subsets, and heterogeneity

Monocytes are named after their morphology: cells with a single nucleus. Human peripheral blood monocytes underwent their developmental stages starting from the myeloid stem/progenitor cell within the BM or spleen to a mature form that enters the bloodstream (11). In the peripheral blood of a healthy individual a normal absolute monocyte cell count (AMC) is 2–8% of white blood cell count; or 200–800 monocytes per microliter of blood. Monocyte numbers may increase (monocytosis) or decrease (monocytopenia) in response to various conditions, such as infection, trauma, systemic inflammatory disorders, autoimmune diseases, (toxic) drug reactions and early BM recovery (12). An underlying neoplastic disorder may be present and therefore when no obvious cause is apparent, a thorough workup and close clinical follow-up is required (13).

**Figure 1:**
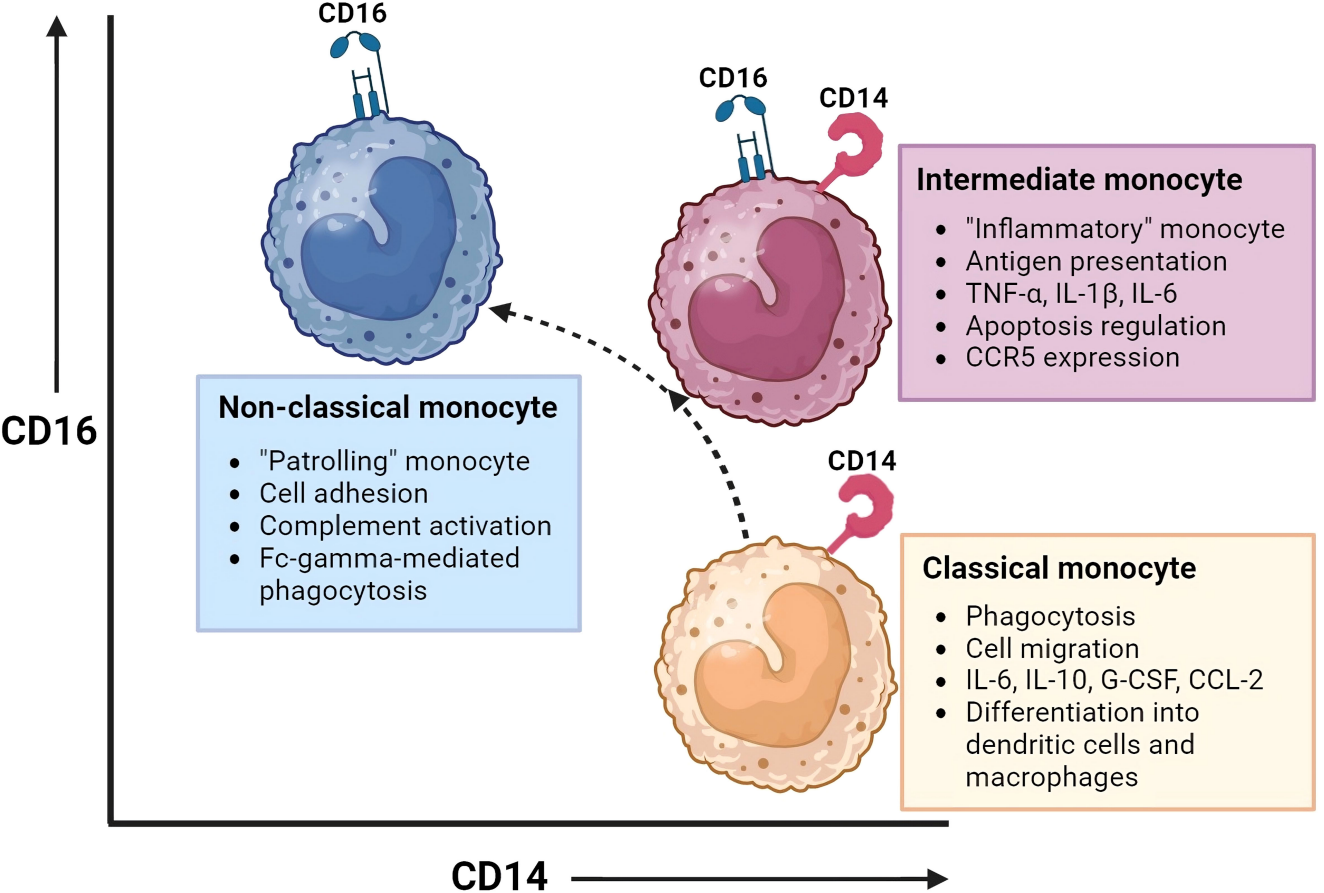
Monocyte subsets and their function. This figure illustrates the monocyte subsets that have been recognized based on the surface expression of CD14 (co-receptor of Toll-Like Receptors) and CD16 (Fc gamma receptor III): classical monocytes (CD14+CD16-), intermediate monocytes (CD14+CD16dim), and non-classical monocytes(CD14dimCD16+). Intermediate and non-classical monocytes may develop from classical monocytes. Each monocyte subset has distinct gene expression and DNA methylation profiles, phagocytic activity, and chemokine receptor expression, resulting in different functions and tissue localization. Created with BioRender.com.

Three monocyte subsets have been recognized based on the surface expression of CD14 (co-receptor of Toll-Like Receptors) and CD16 (Fc gamma receptor III), shown in **Figure 1**. The distribution of these monocyte subsets in peripheral blood is: ~ 85% classical monocytes (CD14+CD16-), ~5% intermediate monocytes (CD14+CD16dim) and ~7% non-classical monocytes (CD14dimCD16+) (11, 14). Classical monocytes are reported to survive for approximately 24h, while intermediate and non-classical monocytes are reported to survive for approximately 4 and 7 days, respectively. A sequential trajectory of monocyte subsets is suggested, where intermediate- and non-classical monocytes are the more mature monocytes that arise from classical monocytes (15–18). With new high-dimensional technologies, such as mass cytometry, more refined subsets may be defined by expression of increased numbers of proteins (19). For example, 6-sulfo LacNac (SLAN) expression was previously used as discriminating marker to define a new monocyte subset. Indeed, heterogeneity at the protein level is observed between the different monocyte subsets. However, there is homogeneity between SLAN+ and SLAN- non-classical monocytes at the transcriptional level. Also, all SLAN+ cells have been shown to be non-classical monocytes. Therefore, SLAN does not show the specificity required to define monocyte cell type or subset (20, 21). While it is important for scientists to adhere to a standardized nomenclature for monocytes to facilitate communication, it is vital to recognize that this framework is subject to evolution. Still, a comprehensive understanding of at times ambiguous protein expression patterns by monocytes remains elusive. Additional research, incorporating epigenetic and metabolomics data, has the potential to enhance our understanding of monocyte function and phenotype, adding additional dimensions to the existing framework.

### 2.2 Monocyte function & erythropoiesis

Each monocyte subset has its own gene expression and DNA methylation profiles, with varying phagocytic activity and chemokine receptor expression patterns (14, 15, 22–24). This results in distinct functions and tissue localization patterns between monocyte subsets. Monocytes have multiple functional properties, such as phagocytosis, antigen presentation, adhesion, cell migration, and production of inflammatory mediators such as cytokines and reactive oxygen species (ROS). All these functions play a role in mediating antitumor, environmental-, and inflammatory responses.

Under steady state, classical monocytes are retained in the BM and spleen and are only dispatched to inflamed tissues when triggered by chemokines. On the other hand, non-classical monocytes are directed to noninflamed tissues as well. While classical and intermediate monocytes rely on CCR2 and their ligands for homing, non-classical monocytes achieve this through CX3CR1 (25). Classical monocytes show superior phagocytic and migratory abilities. In addition, they are characterized by the secretion of high levels of certain cytokines (e.g. IL-6, IL-10, G-CSF and CCL-2) upon LPS stimulation. They are a vital subset with the ability to differentiate into monocyte-derived dendritic cells and macrophages and thereby play an important role in shaping immune responses in peripheral tissues (26). Monocytes transfer their activation state to their progeny which will lead to macrophage polarization towards a pro-inflammatory (M1) or an immune suppressive (M2) profile. Intermediate monocytes, also referred to as the “inflammatory monocytes”, are the only subset that expresses chemokine receptor CCR5. They are specialized in antigen presentation, pro-inflammatory cytokine secretion (e.g. TNF-α, IL-1B, IL-6) and apoptosis regulation. Non-classical monocytes, the “patrolling monocytes”, are known for their adhesive abilities, complement activation and Fc-gamma-mediated phagocytosis (14–18, 22–24, 26).

Under stress, monocytes can exhibit a plasticity that extends beyond their traditional immune functions. For example, monocytes play a remarkable role in stress erythropoiesis, a process crucial for the rapid generation of red blood cells (27). Monocytes are recruited to the spleen, where they contribute to the formation of a specialized niche that supports the expansion and maturation of erythroid progenitor cells. Moreover, the phagocytic capabilities of monocytes clear aging or damaged red blood cells, a process essential for maintaining erythrocyte homeostasis (27). The involvement of monocytes in stress erythropoiesis highlights their versatility.

### 2.3 Monocytes & immune regulation

Monocytes are subject to tight regulation to maintain immune homeostasis (27). Monocytes can adopt diverse functional phenotypes in response to microenvironmental changes, transitioning between pro-inflammatory and anti-inflammatory states. Monocyte plasticity is finely tuned by a complex network of signaling molecules and interactions with other immune cells and is niche-specific. Regulatory mechanisms involve cytokines, such as interferons, interleukins, and chemokines, which influence monocyte recruitment, differentiation and activation. Additionally, toll-like receptors on monocytes recognize pathogen-associated molecular patterns (PAMPs) leading to immune responses. The immune system strives to balance the inflammatory potential of monocytes with mechanisms that prevent excessive tissue damage. The dynamic regulation of monocytes ensures their appropriate involvement in immune defense, tissue repair, and the resolution of inflammation, contributing to the overall effectiveness and balance of the immune system.

### 2.4 Monocytes in healthy, aging individuals

Upon aging, the immune homeostasis changes. Aging is associated with a gradual shift towards a state of persistent, low-grade inflammation, otherwise referred to as ‘inflammaging’ (28). This sustained inflammation may function protectively by increasing immune surveillance to prevent outgrowth of mutated cells. However, it may also have detrimental effects on tissues and organs, contributing to age-related diseases and frailty. Monocytes may add to this process by releasing pro-inflammatory cytokines. Furthermore, mitochondrial dysfunction within monocytes may cause an increase in the release of ROS, further damaging tissues.

Upon inflammaging, sexual disparities become more pronounced. Increased monocyte activity and a more pro-inflammatory state are enriched in elderly males (29–31). This may partially explain why elderly male individuals are more prevalent in the MDS population (32). This emphasizes the importance of the role of monocyte dysfunction in MDS pathogenesis, the need for tailored therapy and the importance of selecting age- and sex- matched healthy controls when investigating the role of monocytes in MDS pathogenesis and leukemic progression.

Inflammation in the hematopoietic niche causes a skewing towards myelopoiesis (33). Persistent inflammation provides a beneficial environment for the outgrowth of malignant myeloid clones due to a higher risk of genotoxic DNA damage (34–37). Several somatic mutations (e.g. DNMT3a, TET2, ASXL1) have been discovered in monocyte stem- and progenitor cells in individuals upon aging. These loss-of-function mutations are associated with clonal hematopoiesis (CH). CH is linked to an increased risk for progression to MDS and AML (2).

## 3 Monocytes in MDS

### 3.1 MDS development and progression

**Figure 2:**
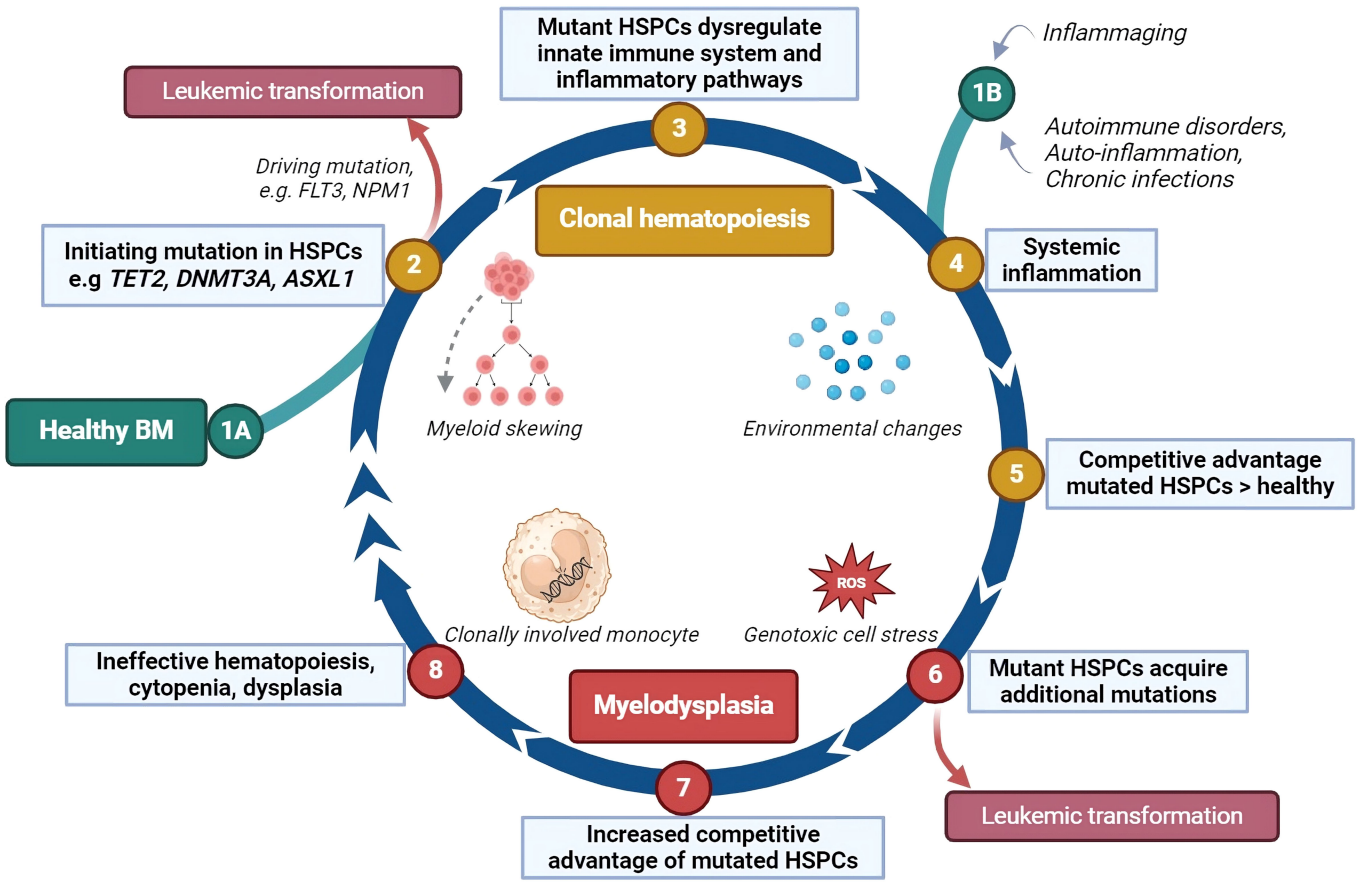
The impact of inflammation on clonal hematopoiesis, myelodysplasia and leukemic transformation. This cyclical model describes how inflammation may contribute to the development of clonal hematopoiesis and its progression to myelodysplasia and leukemia. (1A) Somatic mutations can occur in hematopoietic stem and progenitor cells (HSPCs) by chance, especially as people age, bypassing the DNA repair mechanisms. (2) Some of these mutations may directly lead to leukemic transformation. (3) In other cases, the mutated HSPCs may disrupt inflammatory signaling in the bone marrow (BM), favoring the production of myeloid cells, which enhances the inflammatory response. (4) Systemic inflammation, caused by aging, autoimmune or auto-inflammatory disorders, or chronic infections, increases pro-inflammatory proteins such as cytokines, further driving this cycle. (1B) This may also be the initiating stage of the cyclical model. (5) Mutant HSPCs may respond differently to inflammation, gaining a survival advantage over normal HSPCs, which are suppressed by the inflammatory environment. (6) Cells derived from these mutant HSPCs may be unstable, leading to high cell death rates and the release of stress proteins such as HMGB1 and reactive oxygen species (ROS). This cellular stress can cause additional mutations. (7) New mutations can halt HSPC differentiation, extend their lifespan, or stimulate their growth giving them an even greater competitive advantage and further inhibiting normal HSPC growth and differentiation. (8) The bone marrow becomes ineffective at producing blood cells, leading to cytopenias and dysplasia. The interaction between mutant HSPCs and the immune environment may persist, and about 30% of cases of myelodysplastic syndromes (MDS) eventually progress to leukemia. HSPCs, hematopoietic stem- and progenitor cells; ROS, reactive oxygen species; *FLT3*, fms-related receptor tyrosine kinase 3, an internal tandem duplication of this gene (*FL73*-itd) is among the most frequently occurring leukemic mutations; *NPM1*, nucleophosmin-1, a mutation in this gene is among the most common genetic alterations in acute myeloid leukemia. Created with BioRender.com.

Several models have been developed on the pathogenesis of MDS and its potential transformation into AML. Research on MDS pathogenesis and progression may be roughly divided in two aspects: (1) cell intrinsic changes, including genetic abnormalities; (2) cell extrinsic changes, including changes in immune- and microenvironmental cells and mediators and involves 1) genomic and 2) immunological aspects, respectively. As previously acknowledged, inflammaging encompasses both immune dysregulation and the formation of mutations, which may lead to CH. Additionally, acquired somatic mutations can further influence the skewing of hematopoiesis towards the myelomonocytic lineage or initiate a differentiation block. The hematopoietic cells fail to differentiate adequately, and are prone to die due to their genetic instability, which may be the start of BM failure. The increased production of numerous pro-apoptotic and pro-inflammatory mediators may simultaneously lead to increased apoptosis and pyroptosis of hematopoietic cells. The release of damage associated molecular patterns (DAMPs) upon cell death, together with the release of reactive oxygen species (ROS) in oxidative bursts, further amplifies genotoxic cell stress (34). Acquired loss-of-function or gain-of-function mutations may trigger unrestrained cell growth. The outgrowing clone may be able to create an immune suppressive BM environment, contributing to its superior growth compared to other hematopoietic cells. Accumulation of these events may then result in immune escape and, consequently, leukemic progression (38). A cyclical model of MDS pathogenesis and progression, including both genomic and immunological aspects, is shown in **Figure 2**.

### 3.2 MDS-associated genetic abnormalities and myelomonocytic lineage

Currently, in 80–90% of MDS, mutations are detected by targeted sequencing. Somatic mutations known to cause MDS are mutations in epigenetic regulators, (TET2, DNMT3A, ASXL1, EZH2), RNA spliceosome components (SF3B1, SRSF2, U2AF1), transcription factors (RUNX1, TP53), signal transduction pathways (KRAS, NRAS, JAK2), and the cohesion complex (SMC3, SMC1A, RAD21, STAG2) (39, 40).

The new WHO 2022 criteria have divided MDS entities in two groups, those defined by genetic abnormalities and those defined by morphology (2). All the subtypes within these groups are clinically relevant for prognosis and therapeutic considerations. At present, three types of MDS defined by genetic abnormalities are recognized: MDS with low blasts and isolated 5q deletion (MDS-5q); MDS with low blasts and SF3B1 mutation (SF3B1-MDS); and MDS with biallelic TP53 inactivation (MDS-biTP53). Since not all MDS patients can be classified according to genetic abnormalities yet, the remainder of MDS patients are defined by morphology: MDS with low blast rates (MDS-LB); MDS with fibrosis (MDS-f); MDS with increased blast rates (MDS-IB); and hypoplastic MDS (MDS-h). The latter subtypes are less specific and show the most overlap with other myeloid diseases (41). For example, MDS-IB may be treated according to AML treatment protocols and MDS-h show overlap with other BM failure disorders such as acquired aplastic anemia (AA). In all MDS subtypes, risk assessment is performed, classifying MDS into lower or higher risk of leukemic progression. Recently, the Revised International Prognostic Scoring System for MDS (IPSS-R) has been adapted and now includes molecular information (IPSS-M) (42). The disease outcome in MDS is significantly linked to both the quantity and types of mutations. Multiple mutations are correlated with shorter OS, whereas *SF3B1* mutations are specifically associated with a longer overall survival (OS) (43–45). The integration of molecular data has enhanced the prognostic accuracy of existing risk-stratification schemes in MDS (42).

Currently, prognostic models in MDS do not incorporate immune factors, despite the growing realization of the role of inflammation in CH and MDS development, and the ongoing recognition of immunotherapies as curative treatment option. This gap may be attributed, at least in part, to the observed transcriptional similarities between MDS cancer cells and normal immune cells. Therefore, approaches that quantify the anti-tumor immune response in solid tumors may not be directly applicable in MDS (46) Various strategies and programs have been reported and proposed to bridge this gap (47, 48). The ongoing improvement of technologies, such as single-cell sequencing, in combination with multi-parameter immunophenotyping is essential. Future studies that aim to study inter-tumor heterogeneity in the MDS immune landscape may facilitate a better risk stratification.

### 3.3 Absolute monocyte counts and subsets in MDS

The diagnostic criteria for MDS dictate that the absolute number of PB monocytes should not exceed 0.5x10^9/L, as surpassing this threshold would classify it as chronic myelomonocytic leukemia (CMML) (2). However, within the realm of MDS, the quantity of monocytes, within PB and BM, remains an intriguing biomarker for predicting prognosis and the potential for leukemic transformation.

A lower absolute monocyte count in PB and a higher lymphocyte-to-monocyte ratio (LMR) in PB are both linked to poor survival outcomes (49). However, in a separate study focused on BM monocytes, an observed increase in the percentage of mature monocytes (>6%) in BM correlated with elevated PB monocyte count, a higher IPSS-R score, and a higher BM blast percentage at diagnosis (50). Recently, the apparent contradiction in these data was resolved. It was revealed that the presence of monocytes in MDS follows a U-shaped curve in relation to risk (51). The lowest hazard was observed at around 0.3x10^9/L in PB. MDS with monocytes >0.4x10^9/L was associated with reduced OS, independent of IPSS-R, but not with the risk of transformation to AML. Also, MDS with monocytes <0.2x10^9/L was associated with several adverse disease features, including lower hemoglobin levels, decreased neutrophil and platelet counts, and a higher percentage of BM blasts. Interestingly, this latter group of MDS patients showed a significantly higher risk of progression to AML (51). These data could provide support for a more aggressive therapeutic strategy in patients not identified as clear candidates according to current prognostic scoring systems or recommendations.

**Figure 3:**
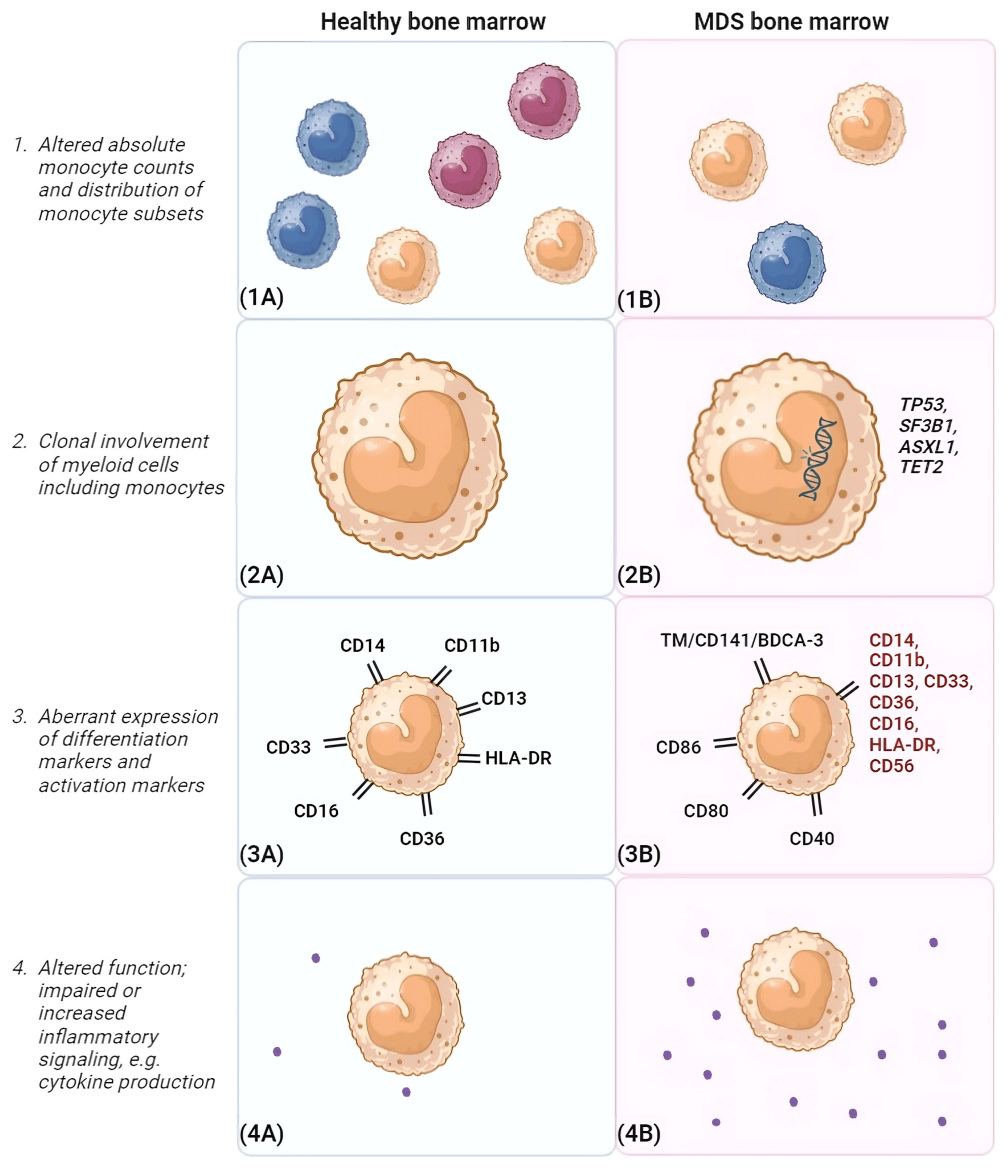
Differences between monocytes in healthy bone marrow and MDS bone marrow. This figure highlights differences that may be observed between monocytes in the bone marrow environment of healthy individuals **(A)** and MDS patients **(B)**. (1) Different monocyte subsets are shown (classical monocytes [orange], intermediate monocytes [green] and non-classical monocytes [blue). In general, frequencies of monocytes are reduced in MDS bone marrow compared to healthy controls. In addition, a disturbance in the distribution of monocyte subsets is observed, favoring classical monocytes. (2) In MDS, monocytes are clonally involved and may harbor mutations similar to those present in the myeloid progenitor cells. Frequently occuring MDS-associated mutations are shown, such as *TP53, SF3B1*, *ASXL1* and *TET2*. (3). In the schematic example of MDS BM, potential aberrancies in differentiation marker expression are highlighted in red. Furthermore, activation markers that can show elevated expression on monocytes in MDS BM are depicted, such as CD86, CD80 and CD40. Also, TM was observed to be elevated on classical monocytes in MDS. (4) In addition, monocytes may be functionally impaired and/or show increased inflammatory signaling. Here, an increased cytokine production in MDS BM compared to healthy BM is shown as example. BM, bone marrow, CD, clusters of differentiation marker; MDS, myelodysplastic neoplasms; TM, thrombomodulin. Created with BioRender.com.

The general distribution of monocyte subsets in PB and BM is similar in MDS and healthy conditions (52). Furthermore, matched PB and BM samples demonstrated that the composition of monocytes in the BM was mirrored in PB (8). However, in higher risk MDS with increased blasts, non-classical monocytes were found to be reduced in MDS BM compared to healthy donor BM (53). Furthermore, an accumulation of classical monocytes in PB distinguishes a subgroup of MDS patients prone to progress towards CMML (7). Likewise, it was observed that MDS patients with BM monocytic proliferation often exhibit CMML-like characteristics (54). In contrast, a link between classical monocytosis in MDS and positive prognostic factors was observed, including elevated white blood cell counts and absolute neutrophil counts (55). Consequently, discerning monocyte subsets is imperative for accurately interpreting the involvement of monocytes in MDS and possible risk assessment (shown in **Figure 3**). A higher percentage of monocytes was associated with lower-risk MDS groups and favorable cytogenetics. An elevated frequency of SF3B1 mutations was present in this MDS subgroup and a tendency towards improved OS was observed (55). Of note, these studies did not include the latest WHO 2022 classification, which may have caused the discrepancies in the observed correlations.

### 3.4 Monocytes and immunophenotypic changes in MDS

In healthy BM with normal hematopoiesis, maturation of the erythroid, megakaryocytic and myelomonocytic lineages follow a certain preserved pattern. This includes an orderly gain and/or subsequent disappearance of cell surface antigens befitting the stage of cell maturation. However, in MDS, hematopoiesis is disturbed in one or more lineages. In addition to numerical changes, aberrancies in the phenotype of monocytes are connected to dysplasia of the maturing myelomonocytic lineage. Lack of or abnormal expression of CD11b, CD13, CD14, CD16, CD33, CD36, HLA-DR and CD56 have been reported (shown in **Figure 3**) (56). These markers are included in a flow cytometric score that has shown to distinguish low-risk MDS from other causes of cytopenias (56). Recently, a positive correlation between the number of aberrantly expressed (dysplasia-associated) surface antigens by monocytes and IPSS-R score was noticed (57). This may indicate that the atypical maturation of monocytes worsens upon disease progression and may be predictive for leukemic transformation.

The expression of various antigens, including the previously mentioned TM, may be up- or down-regulated, influencing the functional state of monocytes. Alternatively, an elevated number of monocytes displaying these antigens potentially influences their microenvironment. In MDS, a rise in markers indicative of activated monocytes has been observed, including CD40, CD80, and CD86 (58). Also, the early upregulation of differentiation markers, such as CD163 and DC-SIGN, has been observed. Notably, an association was found between heightened CD40 expression on monocytes in BM and patients aged below 60 years, as well as those with the cytogenetic abnormality trisomy 8 (58). These markers are considered as predictors for the response to immune suppressive therapy in MDS. The authors hypothesized that CD40 could potentially mirror immunological activity and play a role in guiding therapeutic decisions for these patients (58–60). Some markers have been shown to be co-upregulated on monocytes, like CD40 and CD80 (58). However, activation markers have not been investigated together with markers associated with monocyte dysplasia, consequently no correlations could be made. Future studies are needed to be able to adequately describe how multiple monocyte markers are linked and how they are related to environmental or genomic aberrancies.

MDS subtypes defined by genetic aberrancies have shown to correlate to certain altered immunophenotypes of hematopoietic cells, including monocytes. In SF3B1-MDS, monocytes showed lower expression levels of CD11b, CD36 and CD64 (61). The additional presence of other mutations did not alter the phenotypic features of these monocytes, with exception of a more pronounced decrease in CD64 expression when only *SF3B1* was mutated. Furthermore, the decrease in expression of CD11b and CD36 was more pronounced in cases with the most common *SF3B1* mutation type, K700E, compared to other *SF3B1* mutations (61).

Interestingly, the decrease in CD11b expression by monocytes in SF3B1-MDS correlated to a lower neutrophil count. This is in line with data from a previous study, which showed this for both CD11b and triggering receptor expressed on myeloid cells-1 (TREM-1) on monocytes and neutrophils (62). Hence, the regulatory mechanism of expression of these surface antigens may be similar between monocytes and neutrophils. For example, overexpression of CD56 may be the result of an activated or regenerated BM. Also, CD40 expression may be increased in patients with severe sepsis (63). In addition, it is hypothesized that the alteration in immunophenotype could result directly from a genetic aberrancy in monocytes. In case of an *SF3B1* mutation, it may be the result of alterations in the splicing of surface proteins (61). Alternatively, it may also be triggered by environmental factors associated with SF3B1, like the presence of certain inflammatory cytokines (64). Recently, mutations in SF3B1 have shown to enhance a proinflammatory gene expression profile in blasts (65), confirming the potential relevance of both hypotheses. Further research is needed to elucidate the impact of genetic alterations on monocytic immunophenotype (and function) and the implications for MDS pathogenesis as well as therapeutic strategies.

### 3.5 Monocyte function in MDS

Various monocytic functions related to the inflammatory response triggered by endotoxins have been investigated in MDS. The production of cytokines upon LPS stimulation (e.g. IL-6, TNF-α, IL-10) measured by ELISA and qPCR showed no significant differences compared to healthy controls. Also, phagocytic abilities seemed to be preserved in MDS monocytes, since the phagocytosis of E.Coli particles as assessed by flow cytometry was similar to controls. Monocyte function negatively correlated with blast count, monocyte count and IPSS-R, implying that MDS disease severity affects monocyte function (52). Seemingly in contrast, another study revealed MDS derived monocytes to have reduced phagocytosis activity and low expression of genes involved in immune response triggering, regulation of immune and inflammatory signaling pathways, and a lower response to LPS (66). However, the latter study investigated monocytes derived from 5 patients with CMML or MDS with an excess of blasts according to the WHO 2016. Overall, data from these studies imply that at least part of monocyte functionalities may be retained in low-risk MDS.

The increased expression of MHC-class II (HLA-DR) observed in monocytes in MDS PB and BM (8, 52) may enhance antigen presentation capabilities and subsequent T cell activation. Also, the increased expression of co-stimulatory molecules CD80 and CD86 may enhance the activation of T cells through binding of CD28, which is crucial for the priming of an effective immune response. In addition, CD86 can bind to checkpoint receptor CTLA-4, thereby dampening the immune response and contributing to immune regulation and tolerance. By upregulating specific antigens, monocytes might compensate for immune deficits, such as neutropenia resulting from diseases or as a side effect of treatments like hypomethylating agents and lenalidomide. This compensation may serve as a response to maintain immune functionality. Prospective clinical studies investigating the correlation between monocyte number and/or function and infectious complications are of clinical interest.

Upregulation of CD40 by monocytes has been suggested to be important in the suppression of hematopoiesis in MDS (58). Cell-cell interaction of monocytic CD40 with CD40L on T cells, may result in the monocytic release of pro-apoptotic (e.g. TNF-α, FasL) and pro-inflammatory (cytokines IL-1, IL-6, IL-8, IL-10, IL-12 and chemokine MIP-1a) (co-stimulatory) mediators. In addition, the reported upregulation of a diversity of surface antigens (CD40, CD80, CD86, HLA-DR) on monocytes, will further increase its pro-inflammatory functionality. In PB and BM of low-risk MDS patients, a higher percentage of monocytes expressed CD40, which correlated to a higher percentage of T cells expressing CD40L. Interestingly, stimulating the CD40 receptor by anti-human CD40 in combination with IFN-y resulted in a higher production of TNF-α compared to controls. No differences in TNF-α production were observed between MDS monocytes and controls upon LPS stimulation. Because of the pro-apoptotic properties of TNF-α, these findings were linked to an increased death rate of progenitor cells as one of the causes of suppressed hematopoiesis in MDS (67). This was evidenced by the fact that upon co-culture of BM mononuclear cells with a CD40 blocking antibody (ch5D12), the number of colony forming units significantly increased (58). These findings suggest that assessing CD40 expression on monocytes could delineate a subset of MDS patients in whom immune-mediated hematopoietic failure is essential for the disease process. Furthermore, these data imply that monocytes not simply reflect an ongoing immune attack but are actively contributing to BM failure. Consequently, targeting CD40–CD40L-mediated activation, possibly mediated by pro-inflammatory monocytes, may offer a potential approach to mitigate MDS-related BM failure.

The CD40-CD40L system has proved to be important in various autoimmune diseases, such as rheumatoid arthritis and inflammatory bowel diseases (68–71). Also, in multiple myeloma and non-Hodgkin lymphoma studies have investigated anti-CD40 treatments (72, 73). However, clinical developments were terminated due to severe adverse events. More recently, it was established that exposure to recombinant human soluble CD40 ligand (rh-sCD40L) significantly increased TNF-α mRNA expression and elevated TNF-α protein levels by BM mononuclear cells compared to controls (74). These results suggest that sCD40L plays a role in enhancing TNF-α synthesis at both the mRNA and protein levels, possibly promoting apoptosis in MDS. These observations all point to the CD40/CD40L axis as a viable target for therapeutic intervention in MDS.

Remarkably, the ability to induce macrophages was compromised in MDS-derived monocytes (75). Macrophages induced from PB of MDS patients displayed impaired phagocytic capacity compared to controls. Additionally, macrophages from MDS patients exhibited lower levels of reorganization receptors (CD206 and SIRPα) and impaired phagocytic function. However, there was an increase in the levels of inducible nitric oxide synthase secreted by macrophages in MDS, potentially further contributing to pro-tumorigenic inflammation. Additionally, dendritic cells derived from MDS monocytes exhibited impaired development of dendritic projections and reduced expression of HLA-DR and CD86, suggesting compromised antigen processing and T cell activation capabilities (66). Thus, derivatives from MDS monocytes show impaired function and potentially impaired capabilities to counter the outgrowth of aberrant hematopoietic cells, while maintaining MDS-promoting inflammatory characteristics.

Of note, most of the previously described studies have not distinguished between monocyte subsets. Recently, SLAN+ monocytes demonstrated variations in T-cell skewing in MDS compared to controls. They showed a preference towards supporting T helper 2 expansion (53). Transcriptional analysis revealed altered gene expression, diminished PAMP/DAMP sensing, and defective TLR signaling. Despite intact cytokine secretion, SLAN+ monocytes showed reduced ability to induce T-cell proliferation (53). Future studies are necessary to delineate which monocyte subsets are impaired in MDS and which can show compensatory behavior. Furthermore, monocytes should be examined concurrently with other immune cell populations to be able to understand the cell-cell interactions and to be able to make suggestions for targeted immunotherapies.

## 4 Thrombomodulin expressing monocytes in relation to immune regulation

### 4.1 Thrombomodulin expression by monocytes

In 1991, McCacchren et al. first described that human peripheral blood monocytes expressed thrombomodulin (TM) also known as CD141 or BDCA3 (76). TM is a glycoprotein receptor, transcribed from the *THBD* gene. Its primary substrate is thrombin, a pivotal enzyme in clot formation, which is transformed by TM from procoagulant to anticoagulant (77). TM expression has been shown to be of clinical significance in malignancies (9). Various tumor cells are reported to express TM (78–82). In colorectal and breast cancer, (elevated) expression of TM on tumor cells is associated with a better prognosis (79, 83). In contrast, a recent study on soft tissue sarcomas showed that high levels of TM mRNA were associated with metastasis and poor outcome (81). These data point to different functions of TM in distinct environments.

TM is expressed by various hematopoietic cell types, including myeloid cells such as monocytes, macrophages, dendritic cells, neutrophils and hematopoietic progenitor cells (76, 84, 85). Also, lung epithelial cells (86), vascular smooth muscle cells (87), syncytiotrophoblasts of the placenta (88) and corneal epithelial cells (89) have been reported to express an intracellular and/or extracellular form of TM. Flow cytometry assessments revealed that approximately 18% of healthy classical monocytes in peripheral blood express TM on their surface (8). In the healthy BM, TM expression is relatively low on classical monocytes (~10%, median fluorescence intensity (MFI) 382 ± 65) compared to intermediate (~43%, MFI 1414 ± 245) and non-classical monocytes (~46%, MFI 1279 ± 169) (8). Considering that classical monocytes are the first-stage mature monocyte subset, the increased TM expression on intermediate and non-classical monocytes may indicate that TM expression by monocytes is an acquired phenotypic change upon maturation.

### 4.2 Function of TM receptor on monocytes

Considering the anticoagulant effects of TM, some studies have suggested that TM might dampen the protective role of immunothrombosis and worsen the effects of infection. Immunothrombosis represents a coordinated response of the innate immune system to combat microbial infections by utilizing elements of the coagulation cascade (90). Fibrin networks and clot formation helps to entrap pathogens, limit spread of microbes and provide immune cell support. To investigate this hypothesis, a recent study studied recombinant TM in a rat model including bloodstream infection with methicillin-resistant *Staphylococcus aureus* (MRSA) (91). It was observed that rTM did not in fact promote bacterial burden. Moreover, metabolomics analysis showed a decrease in levels of oxidized glutathione with reference to reduced glutathione (91). Thus, TM may be able to alleviate oxidative stress. This suggests that the expression of TM may protect cells in environments with increased ROS production.

**Figure 4:**
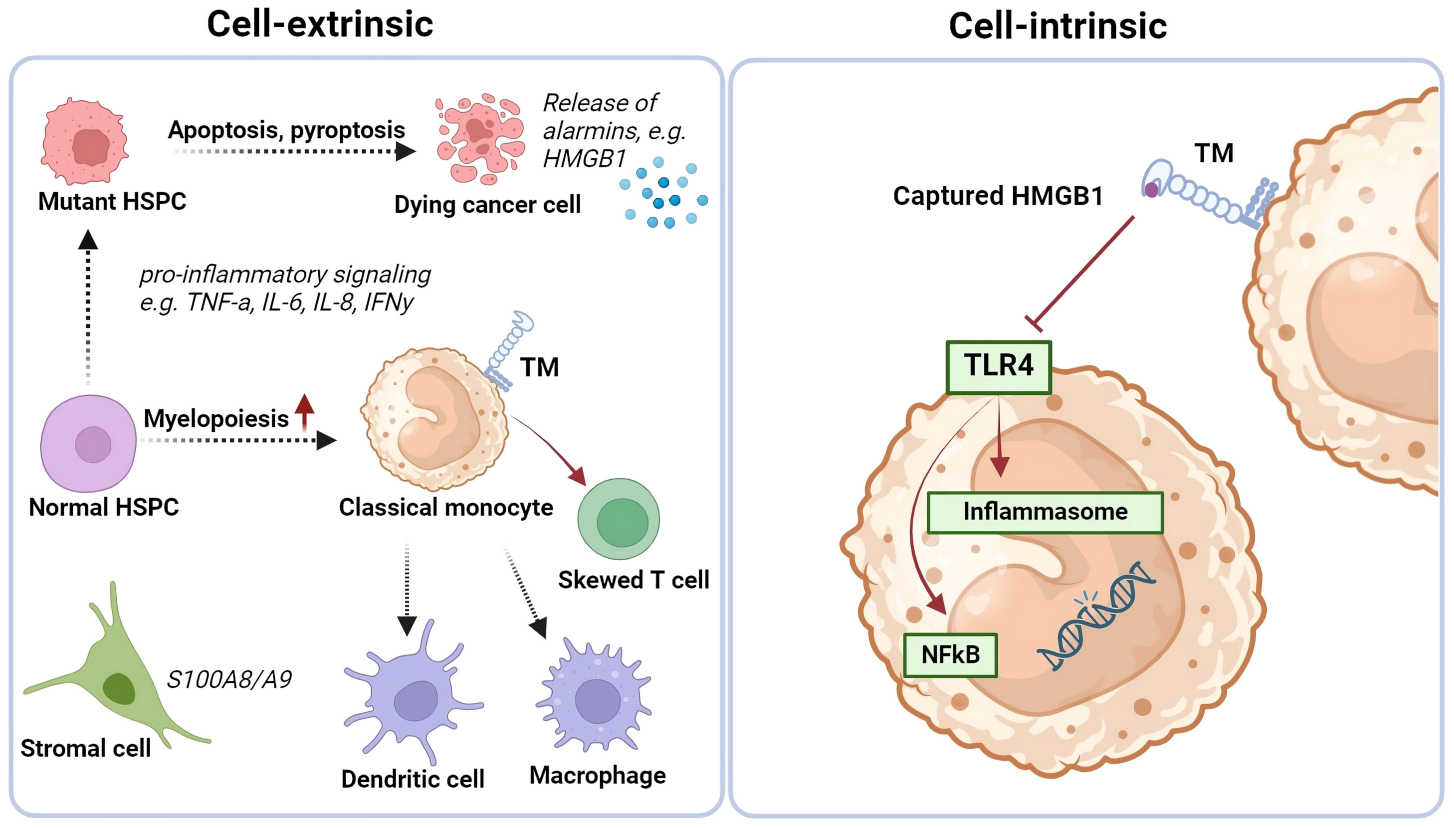
Cell extrinsic and intrinsic changes in MDS BM and (be potential role of TM-expressing monocytes on iflammatory signaling. Thrombomodulin (TM/CD141/BDCA-3) expression is increased on classical monocytes in the bone marrow of some low-risk MDS cases, that have a more pro-inflammatory environment. Monocytes may play a crucial role here by differentiating into antigen-presenting cells, secreting cytokines, sequestering alarmins, and influencing T cell differentiation. The C-type lectin domain of the TM receptor can bind HMGB1, potentially neutralizing its pro-inflammatory effects. TM-expressing monocytes might direct T cells towards an immune-suppressive Th2 and/or T regulatory phenotype. Created with BioRender.com.

One ligand for the C-type lectin domain of TM is HMGB1. HMGB1 is a nuclear protein, either secreted by activated immune cells (e.g., monocytes and macrophages), or passively released by necrotic or damaged cells (92). Once released in the extracellular environment, HMGB1 can bind to cell-surface receptors, such as receptors for advanced glycation end-products (RAGE) and Toll-like-receptor 4 (TLR-4) that may lead to pro-inflammatory NF-κβ pathway activation (92). Furthermore, HMGB1 has been linked to immunothrombosis (90). HMGB1 was shown to trigger the expression and release of tissue factor (TF), thereby exacerbating sepsis caused by gram-negative bacteria following LPS interaction. Additionally, HMGB1 was shown to transport LPS to the cytosol where pyroptosis was induced through activation of caspase-11 (93). The immune regulating function of monocytic TM through binding of HMGB1 has been investigated in two mice models by comparison of primary monocytes to their respective wild-type controls in myeloid-specific TM-deficient mice (LysMcr/TM^flox/flox^) and TM lectin-like domain deleted mice (TM^LeD/LeD^) (94). Extracellular HMGB1 accumulation was enhanced in monocytes from both TM-deficient mice compared to controls (94). These data suggest a role for monocytic TM, especially the C-type lectin domain, in regulating immune processes by neutralizing HMGB1 (shown in **Figure 4**).

Another interesting ligand for the lectin-like domain of TM, in light of monocyte function, is Lewis^y^ (95). This is a carbohydrate that decorates glycoproteins to regulate cellular functions (96). In an inflammatory condition, monocytes are attracted to the site of inflammation by chemokines (97). There, they have to slow down and leave the bloodstream to migrate into tissues, where they can mature into intermediate or non-classical monocytes, or differentiate into dendritic cell populations or macrophages. Monocyte trafficking is under the control of various mechanisms of which monocyte adhesion to endothelial cells is one of its crucial steps (97). TM expressed by monocytic cells from the THP-1 cell line was shown to polarize on the cell surface of monocytes when bound to endothelial cells in a laminar shear flow system (96). This may result in improved migratory capabilities of monocytes. Furthermore, the addition of soluble Lewis^y^ to a suspension of TM-expressing THP-1 cells triggered an increase in phosphorylated p38 mitogen-activated protein kinase (MAPK). Subsequent MAPK signaling may enable adhesion to intercellular adhesion molecule (ICAM)-1 on endothelial cells by activated beta-2 integrins. Indeed, comparison of unmanipulated THP-1 cells with TM knock-down of THP-1 cells (by shRNAi) revealed that TM expressed by THP-1 cells enhanced adhesion to endothelial cells by binding Lewis^y^ (96). These data indicate that TM may influence the immunologic response of monocytes by enhancing cell-cell adhesion and migration of monocytes. The reported functions of monocytic TM, both pro- as well as anti-inflammatory, have been summarized in **Table 1**.

**Table 1:** Summary of inflammatory roles of TM expressed by mononuclear cells.

Reference	Experiment	Cell source	Functional read-out
*Pro-inflammatory role*			
**Ma et al., 2012** (98)	Knock-down TM by shRNAi	Human PBMCs; human THP-1 cells; human U-937 cells; macrophages from LysMcre/TM^flox/flox^ mice	Decreased concentrations of TNF-a, IL-1B, and IL-6 were observed in supernatants of TM-knock down cells
**Lin et al., 2017** (96)	Knock-down TM by shRNAi	Human THP-1 monocytic cells	Enhanced adhesion to endothelial cells (by binding to Lewis-Y on inflamed endothelium) was observed by TM-expressing THP-1 cells.
*Anti-inflammatory role*			
**Cheng et al., 2016** (94)	Knock-down TM by shRNAi	Monocytes from LysMcr/TM^flox/flox^ mice & monocytes from TM^LeD/LeD^ mice	Enhanced accumulation of extracellular HMGB1 was observed in supernatants of monocytes from both knock-down models compared to wild-type controls
**Van Leeuwen-Kerkhoff et al., 2020** (8)	Monocyte and T cell co-cultures	Healthy peripheral blood T cells and TM-expressing monocytes from MDS patients	Polarization of T cells towards Th2 cells, Tregs, and PD-1 expressing clusters of T cells and increased intracellular concentrations of IL-4 and IL-10 in T cells

The functional role of thrombomodulin (TM) expressed by mononuclear cells on the immune system. Both pro- as well as anti-inflammatory functions have been described for TM. ERK, extracellular-signal-regulated kinase; HMGB1, high mobility group box 1; IL, interleukin; LPS, lipopolysaccharide; LysMcre/TM^flox/flox^, mouse model with TM knock-down; MCP-1, Monocyte Chemoattractant Protein 1; MDS, myelodysplastic neoplasm; MMP-9, Matrix metalloproteinase-9; PBMC, peripheral blood mononuclear cells; PD-1, programmed cell death 1; ROS, reactive oxygen species; shRNAi, short hairpin RNA interference; Th2, T helper 2 cells; THP-1, monocyte cell line isolated from the peripheral blood of an acute monocytic leukemia; TNF-a, tumor necrosis factor alpha; TM, thrombomodulin; Tregs, T regulatory cells; U937: a human myeloid leukemia cell line exhibiting monocyte morphology that was derived from a patient with histiocytic lymphoma.

TM may further contribute to immune-suppressive effects of tumor-modulated monocytes, that preferentially differentiate into M2-like macrophages or immature regulatory DCs, through its C-type Lectin domain. TM has been reported on skin-derived CD14+ DCs that induced inflammation-attenuating Tregs (99). Combined with its association with the cross-presenting cDC1 subset (100, 101), this is highly suggestive of a cross-tolerizing ability for TM expressing cDCs. As yet, the functional significance of TM in either cross-presentation or immune suppression remains unclear. Its C-type Lectin domain can down-regulate NF-κB and mitogen-activated protein kinase (MAPK) pathways and might thus interfere with DC and macrophage maturation and drive IL-10 release and Th2 skewing (102, 103). In keeping with this notion, TM expressing blood DCs promote Th2 skewing (104) and *in vitro* generated or skin-derived CD14+TM+ myeloid antigen presenting cells release elevated levels of IL-10 (99, 105). TM can bind Lewis^Y^ glycosylated motifs present on tumor cells through its C-type Lectin domain (95). Similar binding of the C-type Lectin DC-SIGN to differentially glycosylated ligands on cancer cells (e.g. Lewis^X^ and Lewis^Y^ on carcinoembryonic antigen) has been shown to lead to DC suppression and the release of IL-10 (106). In addition, DC-SIGN can modulate TLR-mediated signaling, resulting in prolonged and increased IL-10 transcription (107). It is conceivable that similar processes are at play with TM. Overall, TM expression on monocytes would seem to lead to immune regulatory effects, dampening inflammatory responses and maintaining tolerance and homeostasis.

### 4.3 TM-expressing monocytes in disease

Recently, a relation between TM-expressing monocytes and severe COVID-19 infection was observed (108). Patients with severe disease exhibited a larger fraction of TM-expressing classical monocytes in peripheral blood than controls (flu symptomatic patients that tested negative for SARS-CoV-2 RT-PCR). Also, by single-cell RNA-sequencing analysis of monocytes it was shown that the *THBD* gene was differentially upregulated in severe COVID-19 patients compared to monocytes from mild COVID-19 patients. Pathway analysis of scRNA-seq data highlighted NF-κB activation as a characteristic of monocytes in severe disease. In addition, elevated levels of a crucial component of the canonical NF-κB pathway (phosphorylated transcription factor RelA/p65) were observed in classical monocytes in patients with severe disease in comparison to the control group. Furthermore, in these severe COVID-19 patients, increased levels of IL-6 and S100A8/A9 were detected (108), suggestive for their involvement in the observed upregulation of TM on monocytes. Under these systemic inflammatory conditions TM might have been upregulated in an attempt at immune regulatory feedback.

Also, monocytes are thought to play a key role in amplifying inflammation in patients after cardiac surgery. A higher rate of fever and longer intensive care stay is observed after conventional cardiopulmonary bypass compared to more recent techniques (109). The expression of TM on monocytes was hypothesized to account for this, since its expression was reduced within 30 minutes after conventional cardiopulmonary bypass (110). Also the levels of IL-6 and TNF-α were increased after all cardiac interventions, particularly after conventional cardiopulmonary bypass. Thus, reduced levels of TM expression on monocytes under these conditions was correlated to higher postoperative inflammation, suggesting an anti-inflammatory role (110). Further studies may reveal whether reduction of TM expression by monocytes is a bystander effect or a way of managing the inflammatory environment.

Disseminated intravascular coagulation (DIC) is a disease with systemic fibrin formation, which co-occurs with other diseases such as malignancies (111). In DIC, the proportion of blood monocyte subsets did not show significant differences between overt and non-overt DIC groups, although the expression level of TM by intermediate monocytes was significantly increased in overt DIC patients. Results indicated that a relatively low TM expression by classical and intermediate monocytes correlated to better survival (111). In contrast, a subsequent study by the same group on TM mRNA levels in monocytes demonstrated that low TM mRNA levels were correlated to poor survival in DIC (112). TM mRNA levels were significantly decreased in DIC patients with underlying malignancy or severe infection compared to hepatic failure (112). Whether the observed decrease in TM mRNA expression by monocytes was caused by DIC or the underlying disease remained unclear. More research is necessary to confirm these findings and understand the contrasting survival correlations of TM expression in DIC patients. Potentially, the protein and gene expression of TM do not show a linear correlation, which may explain the contrasting results. Or, the TM receptor shows a delayed folding, which may affect the binding of antibodies. Future studies should take both mRNA as well as surface expression of TM into account.

#### 4.3.1 TM-expressing monocytes in MDS

As previously described, in MDS, an increased percentage of TM-expressing classical monocytes has been discovered in BM as well as in PB (8). No differences were observed regarding TM surface expression by intermediate and non-classical monocytes. Healthy peripheral blood T cells co-cultured with TM-expressing classical monocytes derived from MDS patients, were polarized towards tolerogenic T cells such as T helper 2 cells, T regulatory cells (Tregs), and PD-1 expressing clusters of T cells. Furthermore, these T cells showed increased intracellular concentrations of anti-inflammatory cytokines (IL-4 and IL-10) compared to T cells that were co-cultured with monocytes lacking TM, which mainly showed IFN-y expression (8). These data suggest an anti-inflammatory effect of TM-expressing monocytes derived from MDS patients.

TM-expressing classical monocytes were predominantly found in low-risk MDS (8). Moreover, TM-positivity (>22.5% surface expression measured by flow cytometry as compared to normal) correlated to a better overall and leukemia free survival of MDS patients (8). The BM microenvironment in MDS, particularly in low-risk cases, is characterized by inflammation, raising concerns about immune dysregulation and malignant transformation. When leukemic transformation does occur in low-risk MDS, it is believed to be the result of excessive inflammation and genotoxic cell damage. Since TM-expressing monocytes are correlated to low-risk MDS with relatively good prognosis, we hypothesize that TM’s anti-inflammatory potential may help mitigate excessive immune activation. For example, in some low-risk MDS patients high HMGB1 levels are observed (113), which can induce pro-inflammatory cytokines, thereby contributing to even more immune activation. By neutralizing HMGB1, TM-expressing monocytes may dampen any resulting inflammation and may thus be an interesting target for therapy development in certain low-risk MDS cases.

Notably, percentages of TM-expressing classical monocytes were increased in patients with lower blast counts and in patients with ring sideroblasts (erythroblasts with mitochondrial iron accumulation) (8). By deduction, this suggests an indirect indication of a potential association with a specific MDS subtype, namely SF3B1-MDS. Since TM-expressing monocytes were shown to be clonally involved, identified genetic aberrancies may have a direct effect on monocyte function (8). Studies have shown significant changes in gene expression (e.g. *S100A8* mediator) and pre-mRNA splicing in MDS patients with *SF3B1*-K700E mutations, resulting in the upregulation of pro-inflammatory signaling pathways (65). Additionally, *SF3B1* mutations have been linked to over-activation of nuclear factor kappa B (NF-κB) signaling and increased production of inflammatory cytokines (114). The mRNA of IL-6, an inflammatory cytokine linked to inflammation-associated cancers, was found to be elevated in monocytes from MDS patients with *SF3B1* mutations (115). Furthermore, *SF3B1* mutations can lead to the production of a longer isoform of interleukin-1 receptor-associated kinase 4 (IRAK4), further enhancing NF-κB activation (115). Targeting IRAK4 may in fact offer a potential strategy to alleviate hyperinflammatory features. Overall, *SF3B1* mutations play a significant role in shaping the inflammatory microenvironment in MDS through complex mechanisms (116). Elevated TM expression on the classical monocytes under these conditions may represent a feedback loop and a desperate attempt to combat these mutated SF3B1-associated inflammatory features. Thus, monocyte immunophenotype and function may be influenced by both external and internal factors.

Additional research is required to understand the implications of genetic alterations on monocyte function. Particularly, in SF3B1-MDS, there is a significant decrease in lymphocyte percentages and an increase in erythroid percentages. Hence, it would be intriguing to explore how TM-expressing monocytes affect the BM environment through cytokine release and cell-cell interactions. Investigating the factors that induce TM upregulation on monocytes is necessary to understand the role of cell intrinsic and extrinsic factors in this process. Moreover, exploring the effects of therapies known to target genetic aberrations [such as spliceosome inhibitors (114)] or the microenvironment [such as luspatercept, an inhibitor of transforming growth factor beta that can neutralize negative regulators of late-stage erythropoiesis (117)] in SF3B1-mutated MDS may offer insights into the connection between these factors. Exploring whether hyper-inflammation might be impacted by the presence of TM on monocyte surfaces could provide insights into its potential regulatory role. In conclusion, acquiring additional understanding is vital to grasp the importance of TM expression by monocytes in the context of MDS development and possible treatment approaches.

### 4.4 TM-expressing monocytes as therapeutic target

When considering TM-expressing monocytes as therapeutic target in MDS, both the TM receptor itself as well as features from monocytes could be considered. Recombinant thrombomodulin (rTM) has been suggested for various systemic inflammatory diseases, such as sepsis-associated DIC (118, 119). Furthermore, it has been proposed as a therapeutic strategy for immunothrombosis by inhibiting HMGB1. Such an approach could also be considered to combat genotoxic inflammation in MDS.

Monocyte targeting is possible in several ways, for instance through inhibition of the TNF-α/NF-κβ signaling pathways. The transcription factor NF-κβ, alongside its associated upstream and downstream signaling molecules, play crucial roles in regulating the inflammatory functions of monocytes and macrophages (120). Stimuli known to activate NF-κβ are TNF-α, LPS and IL-1B. Interestingly, it has been shown that TNF-α and LPS can downregulate TM expression on monocytes.

Most studies on human peripheral blood monocytes showed decreased TM mRNA and surface expression upon LPS stimulation, similar to endothelial cells (9). Importantly, subsequent studies showed that this decrease depends on LPS dosage and incubation time (121). Future studies should therefore consider optimal dosage and timing of measurements. In monocytes from TM knock-down models, the production of pro-inflammatory cytokines (TNF-α, IL-1B, IL-6) was reduced upon LPS stimulation (98). This implies that under specific conditions TM on monocytes may also exert pro-inflammatory effects. It was hypothesized that this was the result of an interaction of TM with the CD14/TLR4/MD-2 LPS-binding complex, which actually promoted the inflammatory response to LPS (98). Addition of polymyxin B to inactivate LPS in monocyte cultures revealed no change in the diminished expression of TM. However, when the NF-κβ pathway was obstructed by introduction of MG132 or aurine tricarboxylic acid to monocyte cultures, there was an inhibition of the LPS-induced reduction in TM surface expression (98). These findings imply the involvement of soluble mediators in TM regulation and underscore the crucial role of NF-κβ as a mediator in the suppression of monocytic TM by LPS.

Studies on the response of monocytic TM to TNF-α stimulation show contradictory results. An increase in TM mRNA of peripheral blood monocytes and stable expression on THP-1 cells was reported by two studies of Grey et al. (122, 123) In contrast with these findings, Lin et al. showed that monocytic TM was decreased by TNF-α stimulation (124). Subsequent studies by Lin et al. provided evidence that THP-1 cells responded to TNF-α firstly with a rapid and transient decrease in TM mRNA followed by a sustained and high-level expression (125). Whether these results can be reproduced on peripheral blood monocytes derived from healthy controls and/or patients has yet to be established and might shed light on the feasibility of this approach for therapeutic intervention in MDS.

In MDS with excessive inflammation it would be interesting to counteract the downregulation of TM expressed by monocytes. Therefore, NF-κβ blockers, such as tacrolimus, could be an interesting therapeutic option to enhance the anti-inflammatory potential of TM (126). Additionally, various factors, including TGF-β, IL-1β and oxidized LDL, have been implicated in the downregulation of TM expression on endothelial cells (10). Exploring therapies that counteract (the effect of) these factors could be considered. While there is currently no available data on monocytic TM, a similar mechanism in the up- and downregulation of TM could be assumed for both cell types. In lower-risk MDS, the TGF-β inhibitor luspatercept is currently used for the treatment of severe anemia (127). It promotes erythropoiesis by binding TGF-β which leads to reduced SMAD signaling. The reduction in SMAD results in enhanced maturation of erythrocytes. It would be interesting to investigate whether TM expression on monocytes is also affected in these patients.

It is crucial to recognize that while the immuneregulatory function of TM could potentially mitigate excessive inflammation in low-risk MDS, in high-risk MDS this could lead to an adverse outcome. Therefore, further understanding of the delicate balance within the immune dysregulated environment of MDS is essential for the development of new therapeutic interventions.

## 5 Conclusion

Monocytes are important to maintain immune homeostasis. However, recruited monocytes may also play a role in MDS pathogenesis and progression. In dysregulated environments, monocytes may exhibit aberrant activation and differentiation patterns. Understanding the nuances of monocyte involvement in immune dysregulation is crucial for developing therapeutic strategies that aim to restore immune balance and mitigate the harmful consequences of excessive or impaired immune activity in MDS.

In this review, we hypothesized that TM-expressing monocytes can potentially regulate inflammatory responses in MDS. However, the precise mechanism remains uncertain, requiring further research. It is important to investigate the relationship between clonal involvement and the immune regulatory role of TM-expressing monocytes in MDS. This requires integrating insights from the BM environment and intrinsic cellular abnormalities. Future studies should also explore the association of TM with dysmonopoiesis, co-expression of activation markers, and monocyte function. Advanced technologies such as single-cell RNA sequencing or multi-parameter flow cytometry could aid in achieving these goals and may lead to the identification of novel actionable therapeutic targets.

In summary, TM-expressing monocytes may have a pivotal role in modulating immune activation in MDS, as indicated by their correlation with a subgroup of MDS associated with improved overall and leukemia free survival. However, prolonged immune suppression could have negative consequences on effective immune surveillance, especially in higher-risk MDS cases. Thus, further investigations are imperative to determine whether TM expression merely reflects a bystander effect, constitutes a (failing) negative feedback mechanism, or indeed serves as a potential key regulatory function.

## Author contributions

LJ: Writing – original draft, Conceptualization. NL-K: Conceptualization, Writing – review & editing. TW: Writing – review & editing. TG: Writing – review & editing. AL: Conceptualization, Writing – review & editing.

## References

[1] Lévesque JP Summers KM Millard SM Bisht K Winkler IG Pettit AR. Role of macrophages and phagocytes in orchestrating normal and pathologic hematopoietic niches. Exp Hematol. (2021) 100:12–31.e1. doi: 10.1016/j.exphem.2021.07.001 34298116

[2] Khoury JD Solary E Abla O Akkari Y Alaggio R Apperley JF. The 5th edition of the World Health Organization classification of haematolymphoid tumours: myeloid and histiocytic/dendritic neoplasms. Leukemia. (2022) 36:1703–19. doi: 10.1038/s41375-022-01613-1 PMC9252913 35732831

[3] Hochman MJ DeZern AE. Myelodysplastic syndrome and autoimmune disorders: two sides of the same coin? Lancet Haematol. (2022) 9:e523–34. 10.1016/S2352-3026(22)00138-7 35772431

[4] Adrianzen-Herrera D Sparks AD Singh R Alejos-Castillo D Batra A Glushakow-Smith S. Impact of preexisting autoimmune disease on myelodysplastic syndromes outcomes: a population analysis. Blood Adv. (2023) 7:6913–22. doi: 10.1182/bloodadvances.2023011050 PMC10685168 37729616

[5] Komrokji RS Kulasekararaj A Al Ali NH Kordasti S Bart-Smith E Craig BM. Autoimmune diseases and myelodysplastic syndromes. Am J Hematol. (2016) 91:E280–3. doi: 10.1002/ajh.24333 26875020

[6] Barreyro L Chlon TM Starczynowski DT. Chronic immune response dysregulation in MDS pathogenesis. Blood. (2018) 132:1553–60. doi: 10.1182/blood-2018-03-784116 PMC6182269 30104218

[7] Selimoglu-Buet D Badaoui B Benayoun E Toma A Fenaux P Quesnel B. Accumulation of classical monocytes defines a subgroup of MDS that frequently evolves into CMML. Blood. (2017) 130:832–5. doi: 10.1182/blood-2017-04-779579 28611023

[8] van Leeuwen-Kerkhoff N Westers TM Poddighe PJ de Gruijl TD Kordasti S van de Loosdrecht AA. Thrombomodulin-expressing monocytes are associated with low-risk features in myelodysplastic syndromes and dampen excessive immune activation. Haematologica. (2020) 105:961–71. doi: 10.3324/haematol.2019.219303 PMC7109736 31273091

[9] Loghmani H Conway EM. Exploring traditional and nontraditional roles for thrombomodulin. Blood. (2018) 132:148–58. doi: 10.1182/blood-2017-12-768994 29866818

[10] Conway EM. Thrombomodulin and its role in inflammation. Semin Immunopathol. (2012) 34:107–25. doi: 10.1007/s00281-011-0282-8 21805323

[11] Guilliams M Mildner A Yona S. Developmental and functional heterogeneity of monocytes. Immunity. (2018) 49:595–613. doi: 10.1016/j.immuni.2018.10.005 30332628

[12] Lynch DT Hall J Foucar K. How I investigate monocytosis. Int J Lab Hematol. (2018) 40:107–14. doi: 10.1111/ijlh.12776 29345409

[13] Guglietta S Krieg C. Phenotypic and functional heterogeneity of monocytes in health and cancer in the era of high dimensional technologies. Blood Rev. (2023) 58:101012. doi: 10.1016/j.blre.2022.101012 36114066 PMC12306937

[14] Wong KL Tai JJ Wong WC Han H Sem X Yeap WH. Gene expression profiling reveals the defining features of the classical, intermediate, and nonclassical human monocyte subsets. Blood. (2011) 118:e16–31. doi: 10.1182/blood-2010-12-326355 21653326

[15] Zawada AM Schneider JS Michel AI Rogacev KS Hummel B Krezdorn N. DNA methylation profiling reveals differences in the 3 human monocyte subsets and identifies uremia to induce DNA methylation changes during differentiation. Epigenetics. (2016) 11:259–72. doi: 10.1080/15592294.2016.1158363 PMC4889294 27018948

[16] Tak T van Groenendael R Pickkers P Koenderman L. Monocyte subsets are differentially lost from the circulation during acute inflammation induced by human experimental endotoxemia. J Innate Immun. (2017) 9:464–74. doi: 10.1159/000475665 PMC6738874 28641299

[17] Patel AA Zhang Y Fullerton JN Boelen L Rongvaux A Maini AA. The fate and lifespan of human monocyte subsets in steady state and systemic inflammation. J Exp Med. (2017) 214:1913–23. doi: 10.1084/jem.20170355 PMC5502436 28606987

[18] Tak T Drylewicz J Conemans L de Boer RJ Koenderman L Borghans JAM. Circulatory and maturation kinetics of human monocyte subsets in vivo. Blood. (2017) 130:1474–7. doi: 10.1182/blood-2017-03-771261 28743715

[19] Hamers AAJ Dinh HQ Thomas GD Marcovecchio P Blatchley A Nakao CS. Human monocyte heterogeneity as revealed by high-dimensional mass cytometry. Arterioscler Thromb Vasc Biol. (2019) 39:25–36. doi: 10.1161/ATVBAHA.118.311022 30580568 PMC6697379

[20] Hofer TP Loosdrecht AAvde Stahl-Hennig C Cassatella MA Ziegler-Heitbrock L. 6-sulfo lacNAc (Slan) as a marker for non-classical monocytes. Front Immunol. (2019) 10:2052. doi: 10.3389/fimmu.2019.02052 31572354 PMC6753898

[21] Günther P Cirovic B Baßler K Händler K Becker M Dutertre CA. A rule-based data-informed cellular consensus map of the human mononuclear phagocyte cell space. bioRxiv. (2019). doi: 10.1101/658179

[22] Kapellos TS Bonaguro L Gemünd I Reusch N Saglam A Hinkley ER. Human monocyte subsets and phenotypes in major chronic inflammatory diseases. Front Immunol. (2019) 10:2035. doi: 10.3389/fimmu.2019.02035 31543877 PMC6728754

[23] Gren ST Rasmussen TB Janciauskiene S Håkansson K Gerwien JG Grip O. A single-cell gene-expression profile reveals inter-cellular heterogeneity within human monocyte subsets. PloS One. (2015) 10:e0144351. doi: 10.1371/journal.pone.0144351 26650546 PMC4674153

[24] Ancuta P Liu KY Misra V Wacleche VS Gosselin A Zhou X. Transcriptional profiling reveals developmental relationship and distinct biological functions of CD16+ and CD16- monocyte subsets. BMC Genomics. (2009) 10:403. doi: 10.1186/1471-2164-10-403 19712453 PMC2741492

[25] Dhawan A Padron E. Abnormal monocyte differentiation and function in chronic myelomonocytic leukemia. Curr Opin Hematol. (2022) 29:20–6. doi: 10.1097/MOH.0000000000000689 34854831

[26] Olingy CE Dinh HQ Hedrick CC. Monocyte heterogeneity and functions in cancer. J Leukoc Biol. (2019) 106:309–22. doi: 10.1002/JLB.4RI0818-311R PMC6658332 30776148

[27] Robinson A Han CZ Glass CK Pollard JW. Monocyte regulation in homeostasis and Malignancy. Trends Immunol. (2021) 42:104–19. doi: 10.1016/j.it.2020.12.001 PMC7877795 33446416

[28] Ferrucci L Fabbri E. Inflammageing: chronic inflammation in ageing, cardiovascular disease, and frailty. Nat Rev Cardiol. (2018) 15:505–22. doi: 10.1038/s41569-018-0064-2 PMC6146930 30065258

[29] Olivieri F Marchegiani F Matacchione G Giuliani A Ramini D Fazioli F. Sex/gender-related differences in inflammaging. Mech Ageing Dev. (2023) 211:111792. doi: 10.1016/j.mad.2023.111792 36806605

[30] Márquez EJ Chung CH Marches R Rossi RJ Nehar-Belaid D Eroglu A. Sexual-dimorphism in human immune system aging. Nat Commun. (2020) 11:751. doi: 10.1038/s41467-020-14396-9 32029736 PMC7005316

[31] Jacobsen H Klein SL. Sex differences in immunity to viral infections. Front Immunol. (2021) 12:720952. doi: 10.3389/fimmu.2021.720952 34531867 PMC8438138

[32] Tinsley-Vance SM Ali NA Ball S Aguirre LE Jain AG Hussaini MO. Sex disparities in myelodysplastic syndromes: genotype, phenotype, and outcomes. Clin Lymphoma Myeloma Leuk. (2023) 23:355–9. doi: 10.1016/j.clml.2023.01.007 PMC10121764 36813626

[33] Seyfried AN Maloney JM MacNamara KC. Macrophages orchestrate hematopoietic programs and regulate HSC function during inflammatory stress. Front Immunol. (2020) 11. doi: 10.3389/fimmu.2020.01499 PMC7396643 32849512

[34] Zambetti NA Ping Z Chen S Kenswil KJG Mylona MA Sanders MA. Mesenchymal inflammation drives genotoxic stress in hematopoietic stem cells and predicts disease evolution in human pre-leukemia. Cell Stem Cell. (2016) 19:613–27. doi: 10.1016/j.stem.2016.08.021 27666011

[35] Muto T Walker CS Choi K Hueneman K Smith MA Gul Z. Adaptive response to inflammation contributes to sustained myelopoiesis and confers a competitive advantage in myelodysplastic syndrome HSCs. Nat Immunol. (2020) 21:535–45. doi: 10.1038/s41590-020-0663-z PMC7402480 32313245

[36] Nakad R Schumacher B Damage Response DNA. and immune defense: links and mechanisms. Front Genet. (2016) 7:147. doi: 10.3389/fgene.2016.00147 27555866 PMC4977279

[37] Gañán-Gómez I Wei Y Starczynowski DT Colla S Yang H Cabrero-Calvo M. Deregulation of innate immune and inflammatory signaling in myelodysplastic syndromes. Leukemia. (2015) 29:1458–69. doi: 10.1038/leu.2015.69 PMC4857136 25761935

[38] Gurnari C Visconte V. From bone marrow failure syndromes to VEXAS: Disentangling clonal hematopoiesis, immune system, and molecular drivers. Leuk Res. (2023) 127:107038. doi: 10.1016/j.leukres.2023.107038 36841022

[39] Haferlach T Nagata Y Grossmann V Okuno Y Bacher U Nagae G. Landscape of genetic lesions in 944 patients with myelodysplastic syndromes. Leukemia. (2014) 28:241–7. doi: 10.1038/leu.2013.336 PMC3918868 24220272

[40] Papaemmanuil E Gerstung M Malcovati L Tauro S Gundem G Van Loo P. Clinical and biological implications of driver mutations in myelodysplastic syndromes. Blood. (2013) 122:3616–27. doi: 10.1182/blood-2013-08-518886 PMC3837510 24030381

[41] Tanaka TN Bejar R. MDS overlap disorders and diagnostic boundaries. Blood. (2019) 133:1086–95. doi: 10.1182/blood-2018-10-844670 30670443

[42] Bernard E Tuechler H Greenberg PL Hasserjian RP Arango Ossa JE Nannya Y. Molecular international prognostic scoring system for myelodysplastic syndromes. NEJM Evid. (2022) 1:EVIDoa2200008. doi: 10.1056/EVIDoa2200008 38319256

[43] Bejar R Papaemmanuil E Haferlach T Garcia-Manero G Maciejewski JP Sekeres MA. Somatic mutations in MDS patients are associated with clinical features and predict prognosis independent of the IPSS-R: analysis of combined datasets from the international working group for prognosis in MDS-molecular committee. Blood. (2015) 126:907–7. doi: 10.1182/blood.V126.23.907.907

[44] Bejar R Stevenson K Abdel-Wahab O Galili N Nilsson B Garcia-Manero G. Clinical effect of point mutations in myelodysplastic syndromes. N Engl J Med. (2011) 364:2496–506. doi: 10.1056/NEJMoa1013343 PMC3159042 21714648

[45] Bejar R. Clinical and genetic predictors of prognosis in myelodysplastic syndromes. Haematologica. (2014) 99:956–64. doi: 10.3324/haematol.2013.085217 PMC4040892 24881041

[46] Dufva O Pölönen P Brück O Keränen MAI Klievink J Mehtonen J. Immunogenomic landscape of hematological Malignancies. Cancer Cell. (2020) 38:380–399.e13. 32649887 10.1016/j.ccell.2020.06.002

[47] Winter S Shoaie S Kordasti S Platzbecker U. Integrating the “Immunome” in the stratification of myelodysplastic syndromes and future clinical trial design. J Clin Oncol. (2020) 38:1723–35. doi: 10.1200/JCO.19.01823 32058844

[48] Tentori CA Zhao LP Tinterri B Strange KE Zoldan K Dimopoulos K. Immuno-monitoring of Myelodysplastic Neoplasms: recommendations from the i4MDS Expert panel. Hemasphere. (2024) 8(5). doi: 10.1002/hem3.64s PMC11096644 38756352

[49] Saeed L Patnaik MM Begna KH Al-Kali A Litzow MR Hanson CA. Prognostic relevance of lymphocytopenia, monocytopenia and lymphocyte-to-monocyte ratio in primary myelodysplastic syndromes: a single center experience in 889 patients. Blood Cancer J. (2017) 7:e550. doi: 10.1038/bcj.2017.30 28362440 PMC5380913

[50] Wu A Gao P Wu N Shi C Huang Z Rong C. Elevated mature monocytes in bone marrow accompanied with a higher IPSS-R score predicts a poor prognosis in myelodysplastic syndromes. BMC Cancer. (2021) 21:546. doi: 10.1186/s12885-021-08303-8 33985456 PMC8117396

[51] Silzle T Blum S Kasprzak A Nachtkamp K Rudelius M Hildebrandt B. The absolute monocyte count at diagnosis affects prognosis in myelodysplastic syndromes independently of the IPSS-R risk score. Cancers (Basel). (2023) 15. doi: 10.3390/cancers15143572 PMC10377210 37509235

[52] Pollyea DA Hedin BR O’Connor BP Alper S. Monocyte function in patients with myelodysplastic syndrome. J Leukoc Biol. (2018) 104:641–7. doi: 10.1002/JLB.5AB1017-419RR PMC6113098 29656609

[53] Van Leeuwen-Kerkhoff N Westers TM Poddighe PJ Povoleri GAM Timms JA Kordasti S. Reduced frequencies and functional impairment of dendritic cell subsets and non-classical monocytes in myelodysplastic syndromes. Haematologica. (2022) 107:655–67. doi: 10.3324/haematol.2020.268136 PMC8883570 33567812

[54] Schuler E Frank F Hildebrandt B Betz B Strupp C Rudelius M. Myelodysplastic syndromes without peripheral monocytosis but with evidence of marrow monocytosis share clinical and molecular characteristics with CMML. Leuk Res. (2018) 65:1–4. doi: 10.1016/j.leukres.2017.12.002 29216536

[55] Talati C Zhang L Shaheen G Kuykendall A Ball M Zhang Q. Monocyte subset analysis accurately distinguishes CMML from MDS and is associated with a favorable MDS prognosis. Blood. (2017) 129:1881–3. doi: 10.1182/blood-2016-12-753210 28159734

[56] Duetz C Westers TM van de Loosdrecht AA. Clinical implication of multi-parameter flow cytometry in myelodysplastic syndromes. Pathobiology. (2019) 86:14–23. doi: 10.1159/000490727 30227408 PMC6482988

[57] Li L Yu S Hu X Liu Z Tian X Ren X. Immunophenotypic changes of monocytes in myelodysplastic syndrome and clinical significance. Clin Exp Med. (2023) 23:787–801. doi: 10.1007/s10238-022-00856-7 35916958 PMC9344451

[58] Meers S Kasran A Boon L Lemmens J Ravoet C Boogaerts M. Monocytes are activated in patients with myelodysplastic syndromes and can contribute to bone marrow failure through CD40-CD40L interactions with T helper cells. Leukemia. (2007) 21:2411–9. doi: 10.1038/sj.leu.2404940 17805323

[59] Barrett J Sloand E Young N. Determining which patients with myelodysplastic syndrome will respond to immunosuppressive treatment. Haematologica. (2006) 91:583–4. 16670061

[60] Saunthararajah Y Nakamura R Wesley R Wang QJ Barrett AJ. A simple method to predict response to immunosuppressive therapy in patients with myelodysplastic syndrome. Blood. (2003) 102:3025–7. doi: 10.1182/blood-2002-11-3325 12829603

[61] Duetz C Westers TM In ‘t Hout FEM Cremers EMP Alhan C Venniker-Punt B. Distinct bone marrow immunophenotypic features define the splicing factor 3B subunit 1 (SF3B1)-mutant myelodysplastic syndromes subtype. Br J Haematol. (2021) 193:798–803. doi: 10.1111/bjh.17414 33765355 PMC8252736

[62] Schmidt CS Aranda Lopez P Dopheide JF Schmidt F Theobald M Schild H. Phenotypic and functional characterization of neutrophils and monocytes from patients with myelodysplastic syndrome by flow cytometry. Cell Immunol. (2016) 308:19–26. doi: 10.1016/j.cellimm.2016.07.005 27417453

[63] Sugimoto K Galle C Preiser JC Creteur J Vincent JL Pradier O. Monocyte CD40 expression in severe sepsis. Shock. (2003) 19:24–7. doi: 10.1097/00024382-200301000-00005 12558139

[64] Pollyea DA Harris C Rabe JL Hedin BR De Arras L Katz S. Myelodysplastic syndrome-associated spliceosome gene mutations enhance innate immune signaling. Haematologica. (2019) 104:e388–92. doi: 10.3324/haematol.2018.214155 PMC6717580 30846499

[65] Pollyea DA Kim HM Stevens BM Lee FF Harris C Hedin BR. MDS-associated SF3B1 mutations enhance proinflammatory gene expression in patient blast cells. J Leukoc Biol. (2021) 110:197–205. doi: 10.1002/JLB.6AB0520-318RR 33155727 PMC8612809

[66] Bento LC Bacal NS Rocha FA Severino P Marti LC. Bone marrow monocytes and derived dendritic cells from myelodysplastic patients have functional abnormalities associated with defective response to bacterial infection. J Immunol. (2020) 204:2098–109. doi: 10.4049/jimmunol.1900328 32179638

[67] Parker JE Mufti GJ. The myelodysplastic syndromes: a matter of life or death. Acta Haematol. (2004) 111:78–99. doi: 10.1159/000074488 14646347

[68] Harigai M Hara M Nakazawa S Fukasawa C Ohta S Sugiura T. Ligation of CD40 induced tumor necrosis factor-alpha in rheumatoid arthritis: a novel mechanism of activation of synoviocytes. J Rheumatol. (1999) 26:1035–43. 10332965

[69] Sekine C Yagita H Miyasaka N Okumura K. Expression and function of CD40 in rheumatoid arthritis synovium. J Rheumatol. (1998) 25:1048–53. 9632061

[70] Kasran A Boon L Wortel CH Hogezand RA Schreiber S Goldin E. Safety and tolerability of antagonist anti-human CD40 Mab ch5D12 in patients with moderate to severe Crohn’s disease. Aliment Pharmacol Ther. (2005) 22:111–22. doi: 10.1111/j.1365-2036.2005.02526.x 16011669

[71] Carlsen HS Yamanaka T Scott H Rugtveit J Brandtzaeg P. The proportion of CD40+ mucosal macrophages is increased in inflammatory bowel disease whereas CD40 ligand (CD154)+ T cells are relatively decreased, suggesting differential modulation of these costimulatory molecules in human gut lamina propria. Inflammation Bowel Dis. (2006) 12:1013–24. doi: 10.1097/01.mib.0000234135.43336.72 17075342

[72] Hayashi T Treon SP Hideshima T Tai YT Akiyama M Richardson P. Recombinant humanized anti-CD40 monoclonal antibody triggers autologous antibody-dependent cell-mediated cytotoxicity against multiple myeloma cells. Br J Haematol. (2003) 121:592–6. doi: 10.1046/j.1365-2141.2003.04322.x 12752100

[73] Fanale MA Younes A. Monoclonal antibodies in the treatment of non-hodgkin’s lymphoma. Drugs. (2007) 67:333–50. doi: 10.2165/00003495-200767030-00002 17335294

[74] Iriani A Setiabudy RD Kresno SB Sudoyo AW Bardosono S Rachman A. Expression of mRNA TNFα and level of protein TNFα after exposure sCD40L in bone marrow mononuclear cells of myelodysplastic syndromes. Stem Cell Investig. (2021) 8:6. doi: 10.21037/sci PMC8022284 33829058

[75] Han Y Wang H Shao Z. Monocyte-derived macrophages are impaired in myelodysplastic syndrome. J Immunol Res. (2016) 2016:5479013. doi: 10.1155/2016/5479013 28074192 PMC5198175

[76] McCachren SS Diggs J Weinberg JB Dittman WA. Thrombomodulin expression by human blood monocytes and by human synovial tissue lining macrophages. Blood. (1991) 78:3128–32. doi: 10.1182/blood.V78.12.3128.3128 1660324

[77] Esmon CT Owen WG. The discovery of thrombomodulin. J Thromb Haemost. (2004) 2:209–13. doi: 10.1046/j.1538-7933.2003.00537.x 14995979

[78] Watt J Maguire DG Reid CN Lamont JV Fitzgerald SP Ruddock MW. Thrombomodulin expression in bladder cancer tissue and its association with prognosis and patient survival. Res Rep Urol. (2020) 12:157–65. doi: 10.2147/RRU.S249417 PMC7201128 32432058

[79] Hanly AM Redmond M Winter DC Brophy S Deasy JM Bouchier-Hayes DJ. Thrombomodulin expression in colorectal carcinoma is protective and correlates with survival. Br J Cancer. (2006) 94:1320–5. doi: 10.1038/sj.bjc.6603098 PMC2361416 16622452

[80] Ordóñez NG. Thrombomodulin expression in transitional cell carcinoma. Am J Clin Pathol. (1998) 110:385–90. doi: 10.1093/ajcp/110.3.385 9728615

[81] Asanuma K Nakamura T Asanuma Y Okamoto T Kakimoto T Yada Y. Prognostic Significance of Thrombomodulin mRNA in High-Grade Soft Tissue Sarcomas after 10 years. Orthop Surg. (2020) 12:1726–32. doi: 10.1111/os.12779 PMC7767767 33015987

[82] Tamura A Matsubara O Hirokawa K Aoki N. Detection of thrombomodulin in human lung cancer cells. Am J Pathol. (1993) 142:79–85. 8380956 PMC1886825

[83] Kim SJ Shiba E Ishii H Inoue T Taguchi T Tanji Y. Thrombomodulin is a new biological and prognostic marker for breast cancer: an immunohistochemical study. Anticancer Res. (1997) 17:2319–23. 9216709

[84] Toda M Shao Z Yamaguchi KD Takagi T D’Alessandro-Gabazza CN Taguchi O. Differential gene expression in thrombomodulin (TM; CD141)(+) and TM(-) dendritic cell subsets. PloS One. (2013) 8:e72392. doi: 10.1371/journal.pone.0072392 24009678 PMC3751914

[85] Conway EM Nowakowski B Steiner-Mosonyi M. Human neutrophils synthesize thrombomodulin that does not promote thrombin-dependent protein C activation. Blood. (1992) 80:1254–63. doi: 10.1182/blood.V80.5.1254.1254 1325211

[86] Efremenko AY Seagrave J Clewell HJ Van Landingham C Gentry PR Yager JW. Evaluation of gene expression changes in human primary lung epithelial cells following 24-hr exposures to inorganic arsenic and its methylated metabolites and to arsenic trioxide. Environ Mol Mutagen. (2015) 56:477–90. doi: 10.1002/em.21937 25873331

[87] Tohda G Oida K Okada Y Kosaka S Okada E Takahashi S. Expression of thrombomodulin in atherosclerotic lesions and mitogenic activity of recombinant thrombomodulin in vascular smooth muscle cells. Arterioscler Thromb Vasc Biol. (1998) 18:1861–9. doi: 10.1161/01.ATV.18.12.1861 9848877

[88] Turner RJ Bloemenkamp KW Bruijn JA Baelde HJ. Loss of thrombomodulin in placental dysfunction in preeclampsia. Arterioscler Thromb Vasc Biol. (2016) 36:728–35. doi: 10.1161/ATVBAHA.115.306780 26891741

[89] Huang YH C.C. I Kuo CH Hsu YY Lee FT Shi GY. Thrombomodulin promotes corneal epithelial wound healing. PloS One. (2015) 10:e0122491. doi: 10.1371/journal.pone.0122491 25816372 PMC4376916

[90] Marcos-Jubilar M Lecumberri R Páramo JA. Immunothrombosis: molecular aspects and new therapeutic perspectives. J Clin Med. (2023) 12. doi: 10.3390/jcm12041399 PMC9958829 36835934

[91] Ito T Shrestha B Kakihana Y Maruyama I. Recombinant thrombomodulin alleviates oxidative stress without compromising host resistance to infection in rats infected with methicillin-resistant Staphylococcus aureus. Sci Rep. (2020) 10:17413. doi: 10.1038/s41598-020-74529-4 33060764 PMC7566838

[92] Yuan S Liu Z Xu Z Liu J Zhang J. High mobility group box 1 (HMGB1): a pivotal regulator of hematopoietic Malignancies. J Hematol Oncol. (2020) 13:91. doi: 10.1186/s13045-020-00920-3 32660524 PMC7359022

[93] Lv B Wang H Tang Y Fan Z Xiao X Chen F. High-mobility group box 1 protein induces tissue factor expression in vascular endothelial cells via activation of NF-kappaB and Egr-1. Thromb Haemost. (2009) 102:352–9. 10.1160/TH08-11-0759 PMC2860734 19652887

[94] Cheng TL Lai CH Shieh SJ Jou YB Yeh JL Yang AL. Myeloid thrombomodulin lectin-like domain inhibits osteoclastogenesis and inflammatory bone loss. Sci Rep. (2016) 6:28340. doi: 10.1038/srep28340 27311356 PMC4911607

[95] Wu KK. TM hidden treasure: lectin-like domain. Blood. (2012) 119:1103–4. doi: 10.1182/blood-2011-12-394544 22308281

[96] Lin WL Chen CC Shi GY Ma CY Chang CF Wu HL. Monocytic thrombomodulin promotes cell adhesion through interacting with its ligand, Lewis(y). Immunol Cell Biol. (2017) 95:372–9. doi: 10.1038/icb.2016.110 PMC5415637 27808085

[97] Shi C Pamer EG. Monocyte recruitment during infection and inflammation. Nat Rev Immunol. (2011) 11:762–74. doi: 10.1038/nri3070 PMC3947780 21984070

[98] Ma CY Shi GY Shi CS Kao YC Lin SW Wu HL. Monocytic thrombomodulin triggers LPS- and gram-negative bacteria-induced inflammatory response. J Immunol. (2012) 188:6328–37. doi: 10.4049/jimmunol.1102266 22573811

[99] Chu CC Ali N Karagiannis P Di Meglio P Skowera A Napolitano L. Resident CD141 (BDCA3)+ dendritic cells in human skin produce IL-10 and induce regulatory T cells that suppress skin inflammation. J Exp Med. (2012) 209:935–45. doi: 10.1084/jem.20112583 PMC3348099 22547651

[100] Bachem A Güttler S Hartung E Ebstein F Schaefer M Tannert A. Superior antigen cross-presentation and XCR1 expression define human CD11c+CD141+ cells as homologues of mouse CD8+ dendritic cells. J Exp Med. (2010) 207:1273–81. doi: 10.1084/jem.20100348 PMC2882837 20479115

[101] Jongbloed SL Kassianos AJ McDonald KJ Clark GJ Ju X Angel CE. Human CD141+ (BDCA-3)+ dendritic cells (DCs) represent a unique myeloid DC subset that cross-presents necrotic cell antigens. J Exp Med. (2010) 207:1247–60. doi: 10.1084/jem.20092140 PMC2882828 20479116

[102] Conway EM Pollefeyt S Collen D Steiner-Mosonyi M. The amino terminal lectin-like domain of thrombomodulin is required for constitutive endocytosis. Blood. (1997) 89:652–61. doi: 10.1182/blood.V89.2.652 9002969

[103] Bansal K Sinha AY Ghorpade DS Togarsimalemath SK Patil SA Kaveri SV. Src homology 3-interacting domain of Rv1917c of Mycobacterium tuberculosis induces selective maturation of human dendritic cells by regulating PI3K-MAPK-NF-kappaB signaling and drives Th2 immune responses. J Biol Chem. (2010) 285:36511–22. doi: 10.1074/jbc.M110.158055 PMC2978579 20837474

[104] Yerkovich ST Roponen M Smith ME McKenna K Bosco A Subrata LS. Allergen-enhanced thrombomodulin (blood dendritic cell antigen 3, CD141) expression on dendritic cells is associated with a TH2-skewed immune response. J Allergy Clin Immunol. (2009) 123:209–216.e4. doi: 10.1016/j.jaci.2008.09.009 18947863

[105] Velten FW Duperrier K Bohlender J Metharom P Goerdt S. A gene signature of inhibitory MHC receptors identifies a BDCA3(+) subset of IL-10-induced dendritic cells with reduced allostimulatory capacity in vitro. Eur J Immunol. (2004) 34:2800–11. doi: 10.1002/eji.200324732 15368296

[106] van Gisbergen KP Aarnoudse CA Meijer GA Geijtenbeek TB van Kooyk Y. Dendritic cells recognize tumor-specific glycosylation of carcinoembryonic antigen on colorectal cancer cells through dendritic cell-specific intercellular adhesion molecule-3-grabbing nonintegrin. Cancer Res. (2005) 65:5935–44. doi: 10.1158/0008-5472.CAN-04-4140 15994972

[107] Gringhuis SI den Dunnen J Litjens M van Het Hof B van Kooyk Y Geijtenbeek TB. C-type lectin DC-SIGN modulates Toll-like receptor signaling via Raf-1 kinase-dependent acetylation of transcription factor NF-kappaB. Immunity. (2007) 26:605–16. doi: 10.1016/j.immuni.2007.03.012 17462920

[108] Silvin A Chapuis N Dunsmore G Goubet AG Dubuisson A Derosa L. Elevated calprotectin and abnormal myeloid cell subsets discriminate severe from mild COVID-19. Cell. (2020) 182:1401–1418.e18. doi: 10.1016/j.cell.2020.08.002 32810439 PMC7405878

[109] Laffey JG Boylan JF Cheng DC. The systemic inflammatory response to cardiac surgery: implications for the anesthesiologist. Anesthesiology. (2002) 97:215–52. doi: 10.1097/00000542-200207000-00030 12131125

[110] Tsai CS Tsai YT Lin CY Lin TC Huang GS Hong GJ. Expression of thrombomodulin on monocytes is associated with early outcomes in patients with coronary artery bypass graft surgery. Shock. (2010) 34:31–9. doi: 10.1097/SHK.0b013e3181d494c4 20090566

[111] Hwang SM Kim JE Han KS Kim HK. Thrombomodulin phenotype of a distinct monocyte subtype is an independent prognostic marker for disseminated intravascular coagulation. Crit Care. (2011) 15:R113. doi: 10.1186/cc10139 21489300 PMC3219396

[112] Hong SK Kim JE Han KS Kim HK. Decreased thrombomodulin mRNA expression on peripheral monocytes in disseminated intravascular coagulation patients relates to poor outcomes: the ex vivo effects of lipopolysaccharide and thrombin on monocyte thrombomodulin and CD14 mRNA. Thromb Res. (2013) 132:392–7. doi: 10.1016/j.thromres.2013.07.025 23954259

[113] Velegraki M Papakonstanti E Mavroudi I Psyllaki M Tsatsanis C Oulas A. Impaired clearance of apoptotic cells leads to HMGB1 release in the bone marrow of patients with myelodysplastic syndromes and induces TLR4-mediated cytokine production. Haematologica. (2013) 98:1206–15. doi: 10.3324/haematol.2012.064642 PMC3729900 23403315

[114] Jiang M Chen M Liu Q Jin Z Yang X Zhang W. SF3B1 mutations in myelodysplastic syndromes: A potential therapeutic target for modulating the entire disease process. Front Oncol. (2023) 13:1116438. doi: 10.3389/fonc.2023.1116438 37007111 PMC10063959

[115] Choudhary GS Pellagatti A Agianian B Smith MA Bhagat TD Gordon-Mitchell S. Activation of targetable inflammatory immune signaling is seen in myelodysplastic syndromes with SF3B1 mutations. Elife. (2022) 11. doi: 10.7554/eLife.78136 PMC9427103 36040792

[116] Darman RB Seiler M Agrawal AA Lim KH Peng S Aird D. Cancer-associated SF3B1 hotspot mutations induce cryptic 3’ Splice site selection through use of a different branch point. Cell Rep. (2015) 13:1033–45. doi: 10.1016/j.celrep.2015.09.053 26565915

[117] Kubasch AS Fenaux P Platzbecker U. Development of luspatercept to treat ineffective erythropoiesis. Blood Adv. (2021) 5:1565–75. doi: 10.1182/bloodadvances.2020002177 PMC7948289 33687432

[118] Tamura K Saito H Asakura H Okamoto K Tagawa J Hayakawa T. Recombinant human soluble thrombomodulin (thrombomodulin alfa) to treat disseminated intravascular coagulation in solid tumors: results of a one-arm prospective trial. Int J Clin Oncol. (2015) 20:821–8. doi: 10.1007/s10147-014-0768-1 25385713

[119] Ikezoe T Takeuchi A Chi S Takaoka M Anabuki K Kim T. Effect of recombinant human soluble thrombomodulin on clinical outcomes of patients with coagulopathy after hematopoietic stem cell transplantation. Eur J Haematol. (2013) 91:442–7. doi: 10.1111/ejh.12188 23952647

[120] Mussbacher M Derler M Basílio J Schmid JA. NF-κB in monocytes and macrophages - an inflammatory master regulator in multitalented immune cells. Front Immunol. (2023) 14:1134661. doi: 10.3389/fimmu.2023.1134661 36911661 PMC9995663

[121] Kim HK Kim JE Chung J Kim YT Kang SH Han KS. Lipopolysaccharide down-regulates the thrombomodulin expression of peripheral blood monocytes: effect of serum on thrombomodulin expression in the THP-1 monocytic cell line. Blood Coagul Fibrinolysis. (2007) 18:157–64. doi: 10.1097/MBC.0b013e32801481cb 17287633

[122] Grey ST Hancock WW. A physiologic anti-inflammatory pathway based on thrombomodulin expression and generation of activated protein C by human mononuclear phagocytes. J Immunol. (1996) 156:2256–63. doi: 10.4049/jimmunol.156.6.2256 8690916

[123] Grey ST Csizmadia V Hancock WW. Differential effect of tumor necrosis factor-alpha on thrombomodulin gene expression by human monocytoid (THP-1) cell versus endothelial cells. Int J Hematol. (1998) 67:53–62. doi: 10.1016/S0925-5710(97)00080-7 9594445

[124] Wei HJ Li YH Shi GY Liu SL Chang PC Kuo CH. Thrombomodulin domains attenuate atherosclerosis by inhibiting thrombin-induced endothelial cell activation. Cardiovasc Res. (2011) 92:317–27. doi: 10.1093/cvr/cvr220 21840881

[125] Lin YW Huang CY Shih CM Chang WL Shyue SK Tsai YT. The C-terminal domain of thrombomodulin regulates monocyte migration with interleukin-6 stimulation. Eur J Inflammation. (2014) 12:27–39. doi: 10.1177/1721727X1401200104

[126] Gilmore TD Herscovitch M. Inhibitors of NF-kappaB signaling: 785 and counting. Oncogene. (2006) 25:6887–99. doi: 10.1038/sj.onc.1209982 17072334

[127] Chan O Komrokji RS. Luspatercept in the treatment of lower-risk myelodysplastic syndromes. Future Oncol. (2021) 17:1473–81. doi: 10.2217/fon-2020-1093 33511859

